# Reviewing the Operational Experience on Chemical Looping Combustion of Biomass at CSIC: *i*G‑CLC vs CLOU

**DOI:** 10.1021/acs.energyfuels.4c02880

**Published:** 2024-10-24

**Authors:** Alberto Abad, Luis F. de Diego, Francisco García-Labiano, María T. Izquierdo, Teresa Mendiara, Pilar Gayán, Iñaki Adánez-Rubio, Juan Adánez

**Affiliations:** 120031 Instituto de Carboquímica (ICB-CSIC), Miguel Luesma Castán 4, 50018 Zaragoza, Spain

## Abstract

The unmixed combustion of biomass by chemical looping (Bio-CLC) has outstanding properties to produce energy by thermochemical conversion with CO_2_ capture at low economic and energetic costs as well as minimizing NOx emissions to the atmosphere. A detailed analysis of the experimental results during the biomass combustion in chemical looping combustion (CLC) units is useful to evaluate the Bio-CLC potential for future industrial up-scaling. This work compiles the results obtained during more than 500 h of combustion in the previous years at the Instituto de Carboquímica (ICB-CSIC). Different biomasses were burnt with several oxygen carriers in two singular CLC units. Critical key performance indicators, such as the CO_2_ capture rate and combustion efficiency, have been evaluated considering the combustion mode and the fluidization regime in the fuel reactor. Regarding the combustion mode, results by in situ gasification (*i*G-CLC) vs chemical looping with oxygen uncoupling (CLOU) are compared. As for the fluidization regime, it was bubbling in a 0.5 kW_th_ CLC unit and circulating in a 50 kW_th_ CLC unit. A methodical comparison of the results allows us to understand the fundamentals of the processes and to evaluate the different behaviors observed in each case. The operating conditions having major effects on the Bio-CLC performance were identified, as well as the operating conditions necessary to optimize the CO_2_ capture and the combustion efficiency. In addition, other issues are evaluated, such as the fate of fuel-N in a CLC process, the presence of tar compounds in the CO_2_ stream, or the interaction of ash compounds with the oxygen carrier particles. Finally, possible future actions are discussed to improve the performance of the Bio-CLC process as well as the possible development paths of new oxygen carriers.

## Graphical abstract

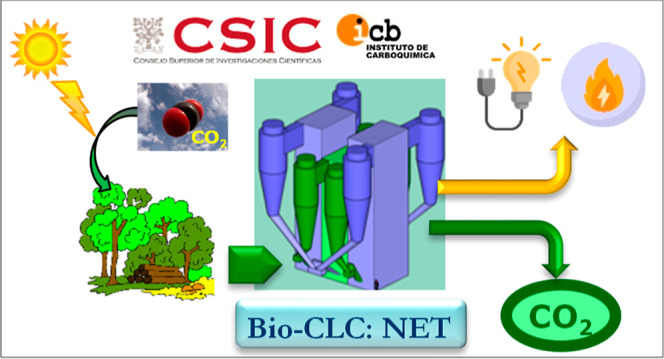

## 1 Introduction

To ensure the access to affordable, reliable, and clean energy is one of the sustainable objective goals (goal 7) defined by the 2030 Agenda for Sustainable Development and promoted by the United Nations (UN).^1^ This is made difficult by the increase in population and economic development, which causes an upward trend in primary energy needs, being increased by 52% in this century.^2^ To guarantee an energy supply, it should be produced from several sources. The energy mix strongly affects the emissions of greenhouse gases (GHGs). Thus, the use of fossil fuels, which have been globally increased by 45% in this century,^2^ is one of the major contributors to the GHG emissions. Indeed, CO_2_ emissions increased by 46%; see Figure 1. These emissions from humans’ activities are unequivocally responsible of the global warming of the planet, with the global surface temperature in 2011–2020 being 1.1 °C higher than in the 1850–1900 period.^3^

**Figure 1:**
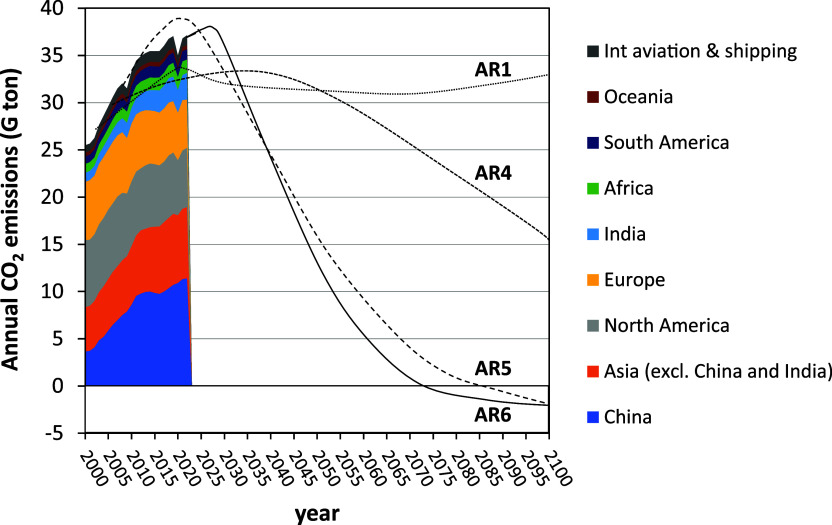
World annual CO_2_ emissions from fossil fuels and industry during this century and forecast of CO_2_ emissions to limit the temperature increase to 1.5 °C by 2100 above preindustrial levels in different reports by the IPCC: Assessment Report (AR1) in 1992,^5^ AR4 in 2007,^6^ AR5 in 2014,^7^ and AR6 in 2023.^3^

Nowadays, there is an international consensus in order to limit anthropogenic CO_2_ emissions to the atmosphere. This compromise was signed by 196 parties at the UN Climate Change Conference (COP21) in Paris.^4^ In this document, the parties undertake to limit “the increase in the global average temperature to well below 2 °C above pre-industrial levels” and pursue efforts “to limit the temperature increase to 1.5 °C above pre-industrial levels”. The Intergovernmental Panel on Climate Change (IPCC) has been making predictions for years on possible CO_2_ emission scenarios, including those scenarios to limit the temperature rise to 2 °C by the end of this century. Among them, Figure 1 includes the prospects for the IS92d scenery in the First AR1 in 1992,^5^ the B1 in the AR4 in 2007,^6^ the RCP2.6 in AR5 in 2014,^7^ and the C3 scenery in AR6 in 2023.^3^ It can be seen that the oldest scenarios were quite undemanding in reducing the emissions of CO_2_. Thus, the most recent reports (AR5 and AR6) show a greater demand for the future reduction of CO_2_ emissions. In all the sceneries, the implementation of carbon dioxide capture and storage (CCS) processes was identified as a key option to achieve the target on CO_2_ emission reduction.^8^ In addition, IPCC scenarios RCP2.6 in AR5 and C3 in AR6 show that in the second half of the 21st century, negative CO_2_ emissions will be necessary; see Figure 1. This means that CO_2_ must be removed from the atmosphere to meet the goal of keeping the increase in the earth’s temperature below 2 °C. In addition, one can expect that CO_2_ emissions will not decrease as it is estimated by the RCP2.6 and C3 scenarios, so the necessity for implementing negative emission technologies (NETs)—also known as carbon dioxide removal (CDR)—will be more necessary, and even earlier, as measures to reduce CO_2_ emissions are postponed over time. NETs focus on the capture of CO_2_ in air (423 ppm average in the last year^9^) and its storage to reduce its atmospheric concentration. CDR pathways include afforestation, reforestation, enhanced weathering, biochar, direct air capture, and bioenergy with carbon capture and storage (BECCS).^10^

The trend followed by North America and Europe shows that energy consumption barely changed in this century. In addition, the decreasing dependence on coal use and the increasing contribution to the energy mix of renewable energies, such as wind, solar, biomass, and wastes, are relevant. Also, the incremental renewable energy is highly relevant in South America, being mostly based on wind and biomass. The increasing use of biomass in the energy mix can offer the opportunity to achieve CDR by using BECCS. BECCS has a high capacity for CO_2_ removal at an acceptable cost,^11^ and it is the only NET for CDR with which, at the same time, we can obtain renewable energy during the CO_2_ capture process.^12^ In fact, the CO_2_ is taken from the atmosphere by plants driven by its photosynthetic function. Other options need to provide energy to a greater or lesser extent.

Commercial CO_2_ capture processes have important economical and energetic costs associated with gas separation steps. In this sense, chemical looping combustion (CLC) highlights as a novel process to capture CO_2_ with low economic and energetic costs for gaseous, liquid, and solid fuels.^13^ In brief, CLC uses a metal oxide (M_*x*_O_*y*_) as an oxygen carrier to supply the oxygen required for fuel combustion. The reduced metal oxide is cyclically regenerated by air. Because the combustion process is split in two steps (redox process: (i) M_*x*_O_*y*_ reduction to M_*x*_O_*y*–1_ to supply oxygen to the fuel and (ii) M_*x*_O_*y*–1_ oxidation by air to M_*x*_O_*y*_), CLC was former proposed to improve the exergy in combustion processes.^14^ Later, it was identified as an alternative combustion method with inherent separation of CO_2_ from the exhaust air because the contact between air and fuel is avoided.^15^

Biomass-based^16^ or organic waste-based^17^ CLC (Bio-CLC) is a BECCS technology which may enable the easy carbon capture for CDR. However, there are some challenges to be addressed before the scale-up and the commercialization of this technology. Sufficient confidence must be had in the use of oxygen carrier materials in Bio-CLC regarding its durability, deactivation trend, or proneness for agglomeration. This fact becomes more relevant when biomass is considered as fuel, which contains a certain number of elements in ash that may cause operating difficulties in the boiler, such as Na, K, Ca, or Si. In addition, the performance of the combustion process should be further understood for a safe design of the reactor that allows maintaining the good qualities of the Bio-CLC process during long periods of operation. Although high CO_2_ capture rates have been reported, complete combustion may not be achieved, which requires a detailed study in order to minimize or avoid the presence of unburnt compounds in the CO_2_ stream.^18^

The objective of this work is to compile results on Bio-CLC and extract relevant conclusions by reviewing and comparing achievements and advancements obtained by the authors from their operational experience on the use of biomass in the CLC process for the BECCS concept. Then, relevant challenges and future prospects for the development of Bio-CLC technology are identified. The authors of this work belong to the Combustion and Gasification Group (at Instituto de Carboquímica: ICB-CSIC) founded and led by Prof. Juan Adánez to whose recognition of his contributions to science the current special issue is dedicated. He focused his research activity mainly on advanced and clean combustion and gasification processes in fluidized bed reactors. Taking into account that coal was, and remains, the world’s largest energy source for power generation, it is not surprising that from the beginning, Prof. Adánez had great interest in coal conversion processes with low SO_2_ and NOx emissions. In addition, he was a pioneer in developing low-emission technologies, anticipating the policies that currently represent the basis for programs such as the UN 2030 Agenda for Sustainable Development or the European Green Deal aiming at an economy with net-zero GHG emissions by 2050 in EU. Thus, Prof. Adánez was interested in R&D activities related to energy or hydrogen production from biomass and CO_2_ capture processes. For the last 20 years, he has had an intense activity in the development of chemical looping technologies for energy and hydrogen production^19^ mainly focused on the advance of oxygen carrier materials, the design and operation of pilot plants, and the process modeling. With coal being the main fossil fuel used for electricity generation, CLC with coal was developed with the objective of reducing CO_2_ emissions by CCS.^20^ Some relevant results from ICB-CSIC on the use of coal were compiled elsewhere.^21^ More recently, the know-how on biomass conversion and CLC was applied to the development of the Bio-CLC process to be applied to the BECCS concept to achieve negative CO_2_ emissions.^18^ This work presents a comprehensive review and analysis of the main results achieved during Bio-CLC operation in pilot plants at the ICB-CSIC.

## 2 Bio-CLC Concept

The use of waste biogenic solid fuels in CLC is highly attractive in future sceneries with restrictions on CO_2_ emissions. Thus, biomass-based CLC (Bio-CLC) has been identified as a promising process to contribute to the BECCS concept with negative CO_2_ emissions.^16,18^ The plants, through photosynthesis, capture CO_2_ from the atmosphere to convert it into organic matter using solar energy. This organic matter may be used as a renewable fuel in the bioenergy concept, with a roughly neutral carbon balance since emitted CO_2_ was previously taken from the atmosphere.^22^ But CDR is achieved if a CCS process is linked to the biomass conversion process, as Figure 2 shows for Bio-CLC.

**Figure 2:**
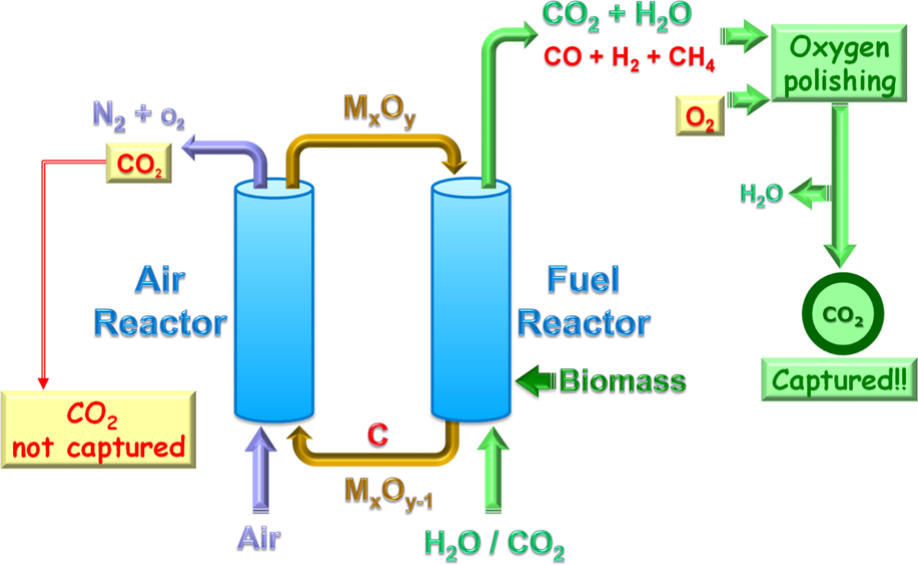
Simplified diagram of the Bio-CLC process.

CLC is based on the use of a metal oxide (M_*x*_O_*y*_) as an oxygen carrier, which transfers the oxygen necessary for the fuel combustion from the air to the fuel, which may be gaseous, liquid, or solid. In a preferred design, a CLC system is composed of two interconnected fluidized bed reactors, the fuel reactor (FR) and the air reactor (AR), and the oxygen carrier circulating between them.^23^ In Bio-CLC, the biomass is fed to the FR of the CLC system, in which it is physically mixed with the oxygen carrier and converted into CO_2_ and H_2_O; see Figure 2.

A scheme of the main processes involved in fuel conversion in the FR is shown in Figure 3a. For that, while biomass is heated to the reactor temperature, it is dried and pyrolyzed to produce volatiles and char, a carbonaceous solid residue; see reaction R[Disp-formula fdR1]. Volatile matter is mainly composed of CO, H_2_, hydrocarbons (mainly CH_4_, C2s, and C3s), CO_2_, H_2_O, and tar compounds. Also, some N and S fuel is emitted together with some volatile elements present in the biomass, such as Na, K, or Cl. Combustible products in volatiles may be oxidized by the oxygen carrier, being the main objective of the Bio-CLC process to achieve a complete conversion to CO_2_ and H_2_O; see reaction R[Disp-formula fdR2]. Char should be gasified—reactions R[Disp-formula fdR3] and R[Disp-formula fdR4]—in order to react with the lattice oxygen in the metal oxide just as it happens with volatile compounds. At the same time, the oxygen carrier is reduced (M_*x*_O_*y*–1_). Because char should be gasified in the FR, this process is usually known as in situ gasification CLC (*i*G-CLC), and mostly oxygen carriers based on iron and manganese oxides have been used.^20^ H_2_O or CO_2_ is supplied to the FR as both fluidization and gasifying agents.

R1 $$biomass\overset{\Delta }{\to }volatiles\left(CO,H_{2},CH_{4},\cdot \cdot \cdot \right)+char\left(C\right)$$

R2 $$CO,H_{2},CH_{4},\cdot \cdot \cdot +nM_{x}O_{y}\to CO_{2}+H_{2}O+nM_{x}O_{y-1}$$

R3 $$char\left(C\right)+H_{2}O\to CO+H_{2}+ash$$

R4 $$char\left(C\right)+CO_{2}\to 2CO+ash$$

**Figure 3:**
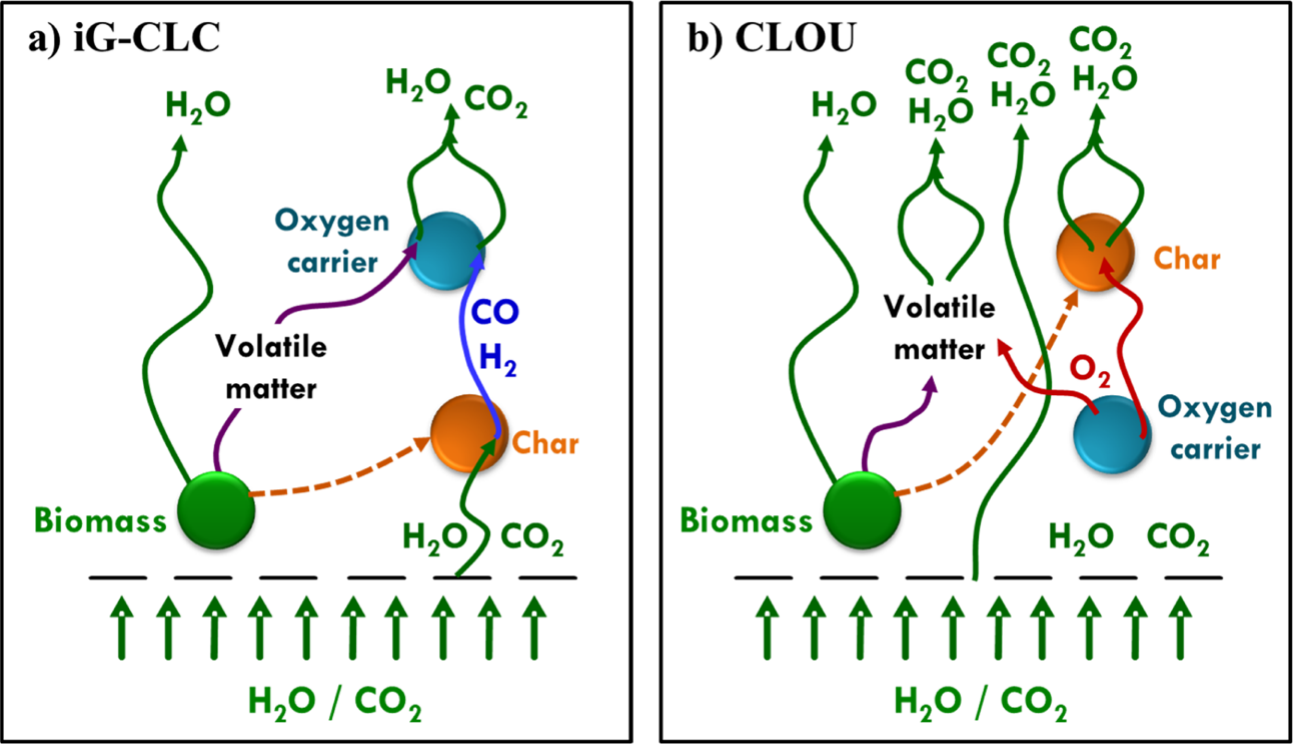
Scheme of the main processes involved in the FR for (a) *i*G-CLC and (b) CLOU with solid fuels.

The oxygen carrier is transported to the AR to be oxidized by air to the initial oxidation state and then starts a new cycle in the FR; see reaction R[Disp-formula fdR5].

R5 $$2M_{x}O_{y-1}+O_{2}\to 2M_{x}O_{y}$$

In order to improve the mechanism through biomass is converted in the FR, Mattisson et al.^24^ proposed the so-called chemical looping with oxygen uncoupling (CLOU) process, where the oxygen carrier is based on a metal oxide with the capability to release gaseous O_2_ in the FR environment; see reaction R[Disp-formula fdR6]. Thus, the solid fuel may be burned with the evolved gaseous oxygen, similar to what happens in combustion by air; see reactions R[Disp-formula fdR7] and R[Disp-formula fdR8] and Figure 3b.

R6 $$2M_{x}O_{y}\to 2M_{x}O_{y-1}+O_{2}$$

R7 $$CO,H_{2},CH_{4},\cdot \cdot \cdot +\left(a+\frac{b}{2}\right)O_{2}\to aCO_{2}+bH_{2}O$$

R8 $$char\left(C\right)+0.5O_{2}\to CO_{2}+ash$$

CLOU requires the use of oxygen carriers based on a metal oxide with the capability of releasing O_2_ at temperatures of interest for combustion, i.e., 800–1000 °C, and later be oxidized by air in the AR. Initially, three metal oxide systems were identified:^24^ CuO/Cu_2_O, Mn_2_O_3_/Mn_3_O_4_, and Co_3_O_4_/CoO. Later, mixed oxides of Mn with other metals (Cu, Fe, Si, Ca, and Mg), perovskite-based materials,^25^ or high-entropy materials prepared by mixing five metal oxides^26^ were identified as materials with suitable thermochemical properties.

In both cases, either *i*G-CLC or CLOU, the net chemical reaction in the global process is the same as that in usual combustion in air with the same combustion enthalpy. Ideally, the complete capture of C-fuel is inherent to this process, as the air does not get mixed with the fuel. A highly pure CO_2_ stream is obtained from the FR after steam condensation, while N_2_ or depleted air is achieved from the AR. However, the feeding of a solid fuel in CLC has three major challenges:

(1) Combustion efficiency: in a fluidized bed, volatile matter is evolved in plumes which has poor contact with oxygen carrier particles, hindering its total conversion to CO_2_ and H_2_O. An oxygen polishing step downstream the FR is proposed to complete gas combustion to CO_2_ and H_2_O.^27^ This option requires the use of highly pure oxygen to avoid the dilution of CO_2_ with N_2_; see Figure 2. Also, some improvements in the FR design or using oxygen carriers with high reactivity or oxygen uncoupling capability can promote the conversion of volatile matter.^28^
(2) CO_2_ capture rate: char must be highly converted in the FR to avoid carbon leaking to the AR where they would be burnt with air. This fact would reduce the CO_2_ capture efficiency because CO_2_ emitted from the AR is not captured. To increase carbon capture, it is necessary to increase char conversion inside the FR. This can be made using a carbon separation system to carry out a selective separation of char particles from the oxygen carrier and their recirculation to the FR. A carbon stripper (CS) has been proposed to carry out the separation of char based on the different fluidizing properties of remaining char and oxygen carrier particles.^29^
(3) The issue of ashes: as a consequence of the ashes present in the solid fuel, it is necessary to drain the ashes from the system to avoid its accumulation in the reactors. The drain stream will also contain some amount of the oxygen carrier. In order to recover the oxygen carrier lost, ash should be separated from oxygen carrier particles. In addition, ash particles can be attached to the oxygen carrier particles, or some elements (Na, K, P, Si,...) diffuse inside these particles promoting agglomeration issues, weakening the structure of the particles, or deactivating the oxygen carrier material. Nevertheless, the issues of ash in the boiler related to fouling and corrosion may be minimized as most of these elements will be concentrated in the stream from the FR. Heat transfer surfaces to extract heat from the AR would not present these types of complications.

There are important differences both in the performance and state of the art of *i*G-CLC and CLOU. Biomass was tested in *i*G-CLC at the 0.1 MW_th_ scale at Chalmers University of Technology with a Mn ore.^30^ In addition, a blend of coal and torrefied biomass was tested with ilmenite at the 1 MW_th_ scale at Darmstadt University of Technology.^31^ The biggest *i*G-CLC unit has been operated under autothermal conditions at the 5 MW_th_ scale at Tsinghua University, but pet coke and lignite were used as fuels in this case.^32^ CLOU development was slower, and it moved from the proof of concept with biomass in 2012 at the 1 kW_th_ scale with a Cu-based oxygen carrier at ICB-CSIC^33^ to the 80 kW_th_ scale with a CaMn-based perovskite at Vienna University of Technology.^34^ Despite the spectacular performances shown by the CLOU process, the use of suitable synthetic materials in CLOU is a drawback vs low-cost materials used in *i*G-CLC.^20^ This work evaluates in depth the main differences between both processes.

## 3 Experimental Section

### 3.1 Lab-Scale CLC Units at the ICB-CSIC

The ICB-CSIC has designed and erected two chemical looping units for processing solid fuels, either in combustion or gasification modes, with nominal powers of 0.5 kW_th_ and 50 kW_th_. Both units were designed to be flexible in its operation, in order to be able to control and measure the solid circulation rate, as well as fixing the temperatures of the reactors by external electrical furnaces. Flexibility is an important characteristic of these units, which allows a detailed evaluation of the effect of operating conditions on process performance.

The 0.5 kW_th_ CLC unit comprises two interconnected fluidized bed reactors, the FR (1:50 mm ID, 200 mm bed height) and the AR (2:80 mm ID, 100 mm bed height) in Figure 4. Both reactors are operated in the bubbling regime, while the riser (3:30 mm ID) above the AR supplies the driving force to support the solid circulation between these reactors. A cyclone at the riser exit (4) recovers the entrained solids to be recirculated to the FR. The solid circulation rate is controlled by varying the opening degree of a solid valve downstream the cyclone (7). On this valve, solids are accumulated (6) to prevent the mixture of gases from reactors. In addition, a diverting solid valve (5) enables the direct measurement of the solid flow rates at any time. Solid fuel is fed with a screw feeder (8) at the bottom of the FR bed and just above the distributor plate. The operation in *i*G-CLC mode is performed fluidizing the FR with H_2_O and/or CO_2_ which also act as gasifying agents. However, in CLOU, the FR was fluidized by N_2_ in most of the cases to evaluate the fuel conversion by the oxygen uncoupling mechanism without interference of char gasification by an external supply of H_2_O/CO_2_. This unit does not have a CS. However, the absence of a CS in this unit facilitates the interpretation of the effect of different operating conditions on the results obtained. More information about this facility can be found in the literature compiled in this work about the operational experience of *i*G-CLC and CLOU; see Table 1.

**Figure 4:**
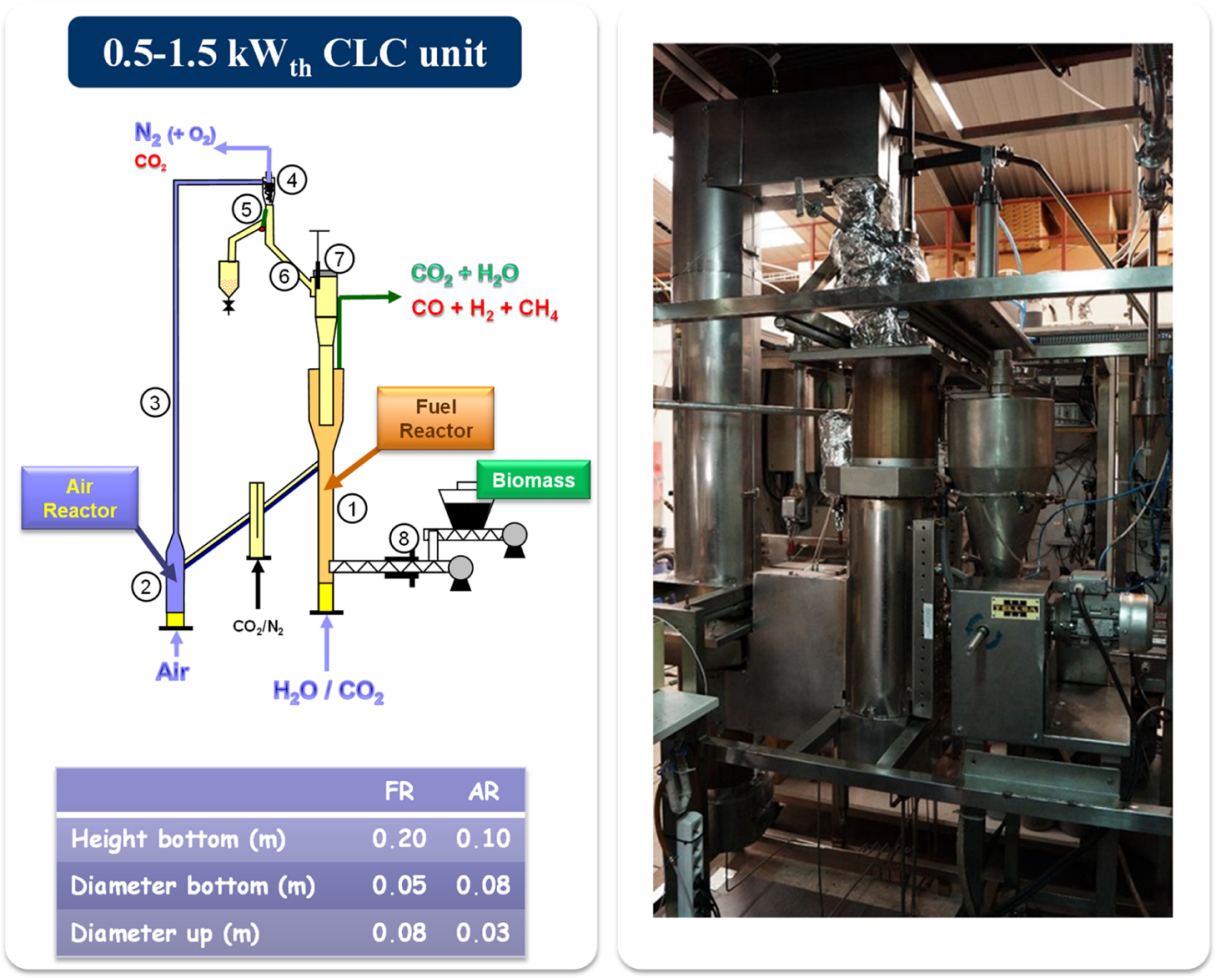
Layout and picture of the 0.5 kW_th_ CLC unit (ICB-CSIC-s1) plant facility for solid fuels.

**Table 1:** Operational Experience (Values Are Combustion Time in Hours) in the 0.5 and 50 kW_th_ CLC Unit with Biomass with Different Oxygen Carriers and Fuels

	mode	pine wood	PFR (pellets)	olive stone	almond shell	swine manure	total (h)
total	*i*G-CLC	86		85	45		216
total	CLOU	159	10	56	46	30	301
total		245	10	141	91	30	517
0.5 kW_th_ unit							
Fe-ore Tierga	*i*G-CLC	62 ^45–47^		25 ^46,47^	25 ^46,47^		112
Mn-ore Gabon	*i*G-CLC	12 ^48^		10 ^48^	10 ^48^		32
Mn-ore S. Afr.	*i*G-CLC	12 ^48^		10 ^48^	10 ^48^		32
60CuO/MgAl_2_O_4_	CLOU	10 ^33,49,50^					10
30CuO/MnFe	CLOU	23 ^51,52^		7 ^51^	7 ^51^		37
30CuO/MnFe/Kao	CLOU	74 ^52,53^		14 ^53^	14 ^53^	30 ^54^	132
Cu34Mn66	CLOU	30 ^47,49,55^		25 ^47,49,55^	25 ^47,49,55^		80
Mn66FeTi7	CLaOU	12 ^56^					12
TOTAL 0.5 kW_th_ unit		235		91	91	30	447
50 kW_th_ unit							
Fe-ore Tierga	*i*G-CLC			40 ^57^			40
30CuO/MnFe	CLOU	10 ^36^	10 ^36^	10 ^36^			30
TOTAL 50 kW_th_ unit		10	10	50			70

The 20–50 kW_th_ CLC unit was designed to operate in both *i*G-CLC and CLOU modes. The nominal thermal power is 20 kW_th_ for *i*G-CLC and 50 kW_th_ for CLOU. The difference of nominal power is because of the better performance of CLOU with respect to *i*G-CLC on the basis of CO_2_ capture and combustion efficiency.^35^ The maximum nominal power for CLOU has been recently achieved with coal as a fuel.^36^

This unit is based on two interconnected circulating fluidized bed reactors, being the FR and AR; see Figure 5. Also, a CS is included in order to minimize the flow of char entering to the AR. The CS was designed to separate char particles from oxygen carrier particles due to differences in its terminal velocity. Thus, successful separation is achieved with small fuel particles,^37^ but it is not useful when pelletized biomass is fed.^38^ A double loop seal below the FR cyclone allows a stable solid circulation regardless of the gas velocity or solid amount in the reactors. The measurement of the solid circulation rate is performed by means of two diverting solids devices located downstream of the FR and AR cyclones. The solid fuel was fed through a screw feeder system just above the distributor plate in the FR. It is intended to convert the fuel to CO_2_ and H_2_O, minimizing the presence of unburnt compounds.

**Figure 5:**
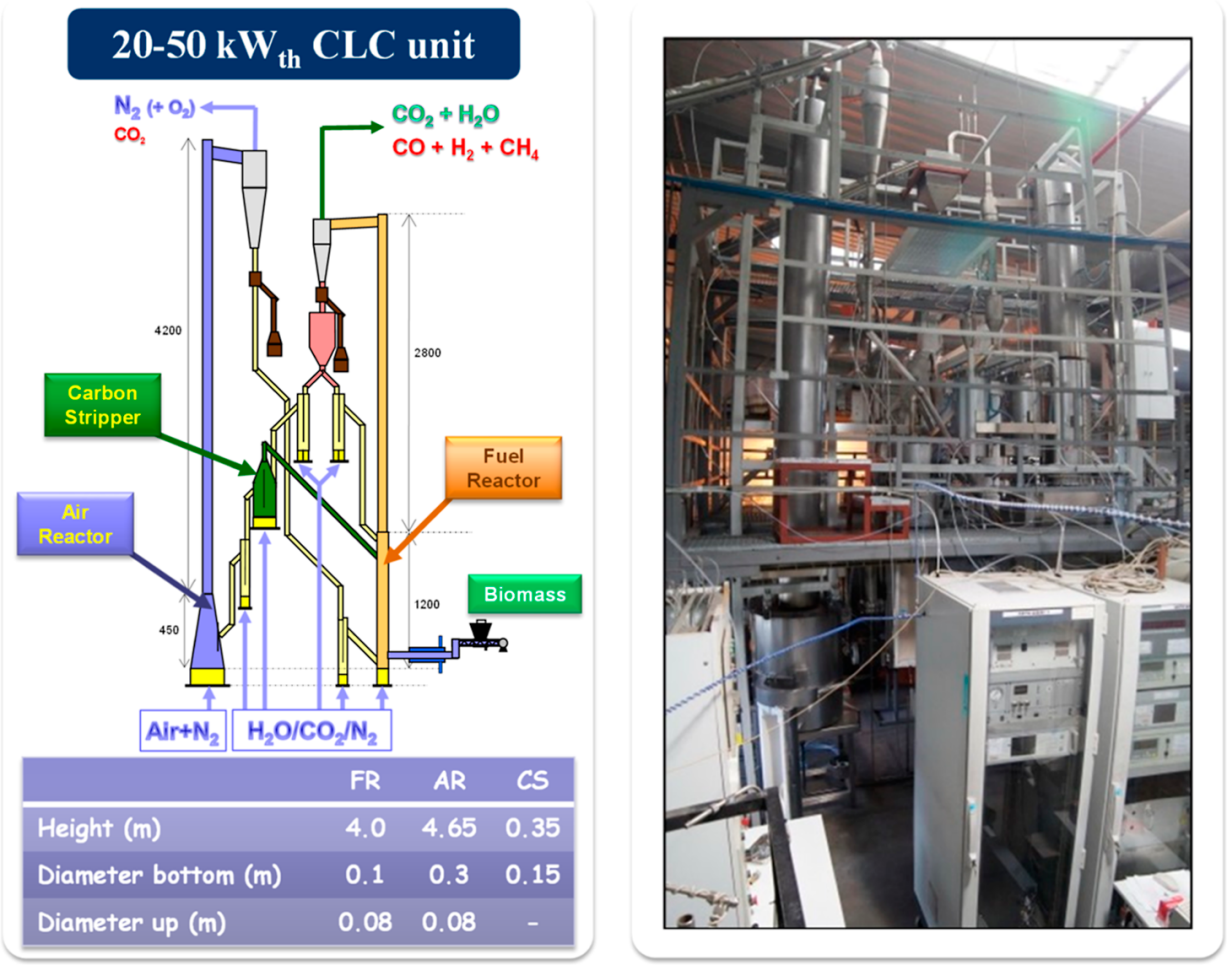
Layout and picture of the 50 kW_th_ CLC unit (ICB-CSIC-s50) plant facility for solid fuels.

The dimensions of the reactors, shown in Figure 5, are defined by the design values of solids inventory and gas velocity. The height of AR and FR reactors was determined in order to arrange all the elements of the CLC unit. Thus, the AR height is 4.65 m, whereas the FR is 4.0 m tall. The FR includes a bottom section (1.2 m high) with low gas velocity (∼1 m/s) which operates in the bubbling/slag regime. The FR upper part (2.8 m high) operates at the turbulent/fast fluidization regime as the velocity increases to 4–6 m/s. The introduction of internals in the riser improved the combustion efficiency in the FR^39^ due to solids accumulation on each constriction.^40^ However, these solids caused a high pressure drop in the FR, which made the maintenance of adequate operation difficult due to the decompensation of pressures between different parts of the unit (AR-CS and AR-FR) that sometimes could not be compensated by the presence of the loop seals. Therefore, tests with biomass were carried out with an FR riser without constrictions. A former design of this unit included an oversized AR^35^ which required the need for a high amount of solids (∼100 kg) that slowed down the achievement of steady-state operation.^41^ Later, the AR volume was decreased, which facilitated rapid steady-state operation with 40–50 kg of solids in the whole unit.^38^ More information about the characteristics and the operational experience with biomass in this unit can be found in the literature cited in Table 1. The main results are compared and discussed in this work. Most of the operational experience was achieved in *i*G-CLC mode, but some relevant results in CLOU mode in the 20–50 kW_th_ interval will be also anticipated here. In both cases, the FR was fluidized by H_2_O or CO_2_, which acts as a fluidization medium and gasification agent.

**Table 2:** Main Properties of the Oxygen Carriers Used in the 0.5 and 50 kW_th_ CLC Units with Biomass

	Fe-ore Tierga	Mn-ore Gabon	Mn-ore S.Afr.	60CuO/MgAl_2_O_4_	30CuO/MnFe	30CuO/MnFe/Kao	Cu34Mn66	Mn66FeTi7
oxygen transport capacity (%)	2.5	5.1	4.7	6.0	2.0	2.3	4.0	9.5
crushing strength (N)	5.8	1.8	4.6	2.4	2.2	1.8	1.9	1.9
porosity (%)	26.3	38.7	12.3	16.1	51.6	29.5	12.0	30.
skeletal density (kg/m^3^)	4216	2800	3510	4600	5125	4720	4100	4939

There are some critical differences between the 0.5 and 50 kW_th_ CLC units that can affect the process performance and the interpretation of the results, namely,

- The FR fluid dynamics and solid flow: the FR in the 0.5 kW_th_ CLC unit operates at the bubbling regime, while the 50 kW_th_ CLC unit operates as a circulating fluidized bed reactor. In addition, the specific solids inventory per unit of thermal power in the 0.5 kW_th_ CLC unit (500–2000 kg/MW_th_) is higher than in the 50 kW_th_ unit (100–500 kg/MW_th_). This difference affects the gas–solid contact and the mean solids residence time and therefore may have influence on the combustion efficiency and char conversion.
- In the 0.5 kW_th_ CLC unit, the solids output flow from the FR is by overflow and driven by gravity, while solids in the 50 kW_th_ are entrained upward and separated from gases by a cyclone. This difference should be considered since the existence of unconverted char particles escaping with gases from the FR may affect the interpretation of results concerning the carbon balance, e.g., to the char conversion in the FR and CO_2_ capture rate. This fact is highlighted in the evaluation of the key performance indicators (KPIs).

### 3.2 KPIs in Bio-CLC

The analysis of the *i*G-CLC and CLOU operational experience in both CLC units with biomass is mainly performed by calculating the CO_2_ capture rate and combustion efficiency of the system. Additionally, other parameters are of help to deepen the understanding of the processes happening in the reactors of the CLC units, such as the fuel conversion or the char conversion in the FR. A comprehensive relation between these KPIs and the operation parameters is shown in Figure 6, and it will be considered in the Results section to evaluate the KPI values as a function of the operation parameters or the CLC unit characteristics. These parameters are calculated by carrying out detailed mass balances for C and O to the system. Here, a conceptual expression for all parameters is shown in each equation. For detailed equations to be applied with experimental results from the operation of CLC units, readers are referred to the articles where the results are published (Table 1) or to a previous review focused on CLC with solid fuels.^20^ So, the KPIs are defined as follows:

- Fuel conversion, *X*_f_: fuel conversion evaluates the fraction of the fuel being converted in the CLC unit. Assuming that most of volatile matter—which includes elements such as C, H, and O—is released in the FR, and char will be mostly composed by C; unconverted fuel would correspond to unconverted carbon in the unit. Therefore, the fuel conversion may be evaluated by a carbon balance considering the carbon present in the gases released from the FR and AR in relation to the carbon in the fuel fed to the CLC unit. Note that carbon nonconverted in the CLC unit is assumed to escape with gases from the FR as unconverted char or being accumulated somewhere in the unit.

1 $$X_{f}=\frac{carbon\;from\;fuel\;in\;gaseous\;compounds\;from\;the\;FR\;\text{and}\;AR}{carbon\;in\;the\;fuel\;fed}=\frac{carbon\;in\;the\;fuel\;fed-unconverted\;carbon}{carbon\;in\;the\;fuel\;fed}$$

- Char conversion in the FR, *X*_*char*_: both in *i*G-CLC and CLOU, it is intended that carbon in char was mostly converted in the FR. Thus, the carbon in char converted in the FR may be evaluated by the char conversion defined by eq 2.

2 $$X_{char}=\frac{carbon\;from\;char\;converted\;in\;the\;FR}{fixed\;carbon\;in\;the\;fuel\;fed}$$

- In the 0.5 kW_th_ CLC unit, the char conversion defined by eq 2 is affected by the unconverted char escaping with gases from the FR as it has not been able to be converted before being elutriated. To consider this collateral effect on the evaluation of the results, char conversion has been redefined considering only the effective carbon in char, *X*_char,eff_, which discounts the unconverted carbon escaping with gases from the FR to the fed carbon; see eq 3. In this way, it is feasible both to calculate the estimated CO_2_ capture rate in the case that carbon loss in unconverted char was avoided^42^ and to evaluate the char conversion rate as a function of the residence time of char in the FR, being different to the mean residence time of oxygen carrier particles in the FR.^43^

3 $$X_{char,eff}=\frac{carbon\;from\;char\;converted\;in\;the\;FR}{fixed\;carbon\;converted\;in\;the\;whole\;CLC\;unit}$$

- CO_2_ capture rate, η_CC_: it is usually considered that all carbon in gases from the FR is captured regardless of whether it was in the form of CO_2_, CO, CH_4_, or other hydrocarbons. This is so since the use of an oxygen polishing step downstream the FR is proposed to achieve complete oxidation to CO_2_ and H_2_O of the gases that have not been converted into the FR. Thus, the noncaptured CO_2_ is that exiting from the AR, and the conventional definition of the CO_2_ capture rate is presented in eq 4.

4 $$\eta_{CC}=\frac{carbon\;from\;fuel\;in\;gases\;from\;the\;FR}{carbon\;in\;the\;fuel\;fed}=1-\frac{carbon\;from\;fuel\;in\;gases\;from\;the\;AR}{carbon\;in\;the\;fuel\;fed}$$

- Although the carbon balance in the AR is easier than in the FR, the existence of unconverted char escaping with gases from the FR makes the calculated CO_2_ capture rate was different being calculated using either carbon in gases from the FR or AR. Thus, if solid carbon scaping in gases was avoided, it would be burned to CO_2_ in the AR, modifying the flow of “carbon in gases from AR” in eq 4. Therefore, the use of “carbon in gases from FR” is preferred. To circumvent the inherent difficulty of the carbon balance in the FR, the use of the oxide oxygen fraction, η_OO_ ^44^ (also named as χ_oo_ in ref 20), has been proposed; see eq 5. This parameter is based on an oxygen balance in the AR which is much easier and with less uncertainty. It relates the oxygen transferred to the fuel by the oxygen carrier with the total oxygen reacted in the AR, which also includes oxygen reacted with bypassed carbon to the AR. Although the η_OO_ parameter is not based on the strict definition of CO_2_ capture, it is a good indication of this parameter at low carbon flows in the AR, i.e., high CO_2_ capture values. However, this value may differ from the actual CO_2_ capture value at lower CO_2_ capture values since the oxygen demanded by carbon is different to carbon demanded by the fuel.^44^

5 $$\eta_{oo}=\frac{oxygen\;reacted\;in\;the\;AR\;only\;with\;the\;oxygen\;carrier}{total\;oxygen\;reacted\;in\;the\;AR}$$

- Because the unconverted char escaping with gases from the FR can mislead the interpretation of the CO_2_ capture in the 0.5 kW_th_ CLC unit, an effective CO_2_ capture defined by eq 6 is usually used in this unit.^20^ Thus, CO_2_ capture is calculated taking into account only carbon in gases from the FR and AR.

6 $$\eta_{CC,eff}=\frac{carbon\;from\;fuel\;in\;gases\;from\;the\;AR}{carbon\;from\;fuel\;in\;gases\;from\;the\;AR\;\text{and}\;FR}$$

- Combustion efficiency, η_comb_: this parameter evaluates the combustion degree of the fuel in the FR. It is calculated considering the oxygen required to oxidize unconverted gases from the FR to CO_2_ and H_2_O.

7 $$\eta_{comb}=1-\Omega_{OD}=1-\frac{oxygen\;demanded\;\text{for}\;the\;complete\;combustion\;of\;gases\;from\;the\;FR}{oxygen\;demanded\;\text{for}\;the\;complete\;combustion\;of\;the\;fuel}$$

**Figure 6:**
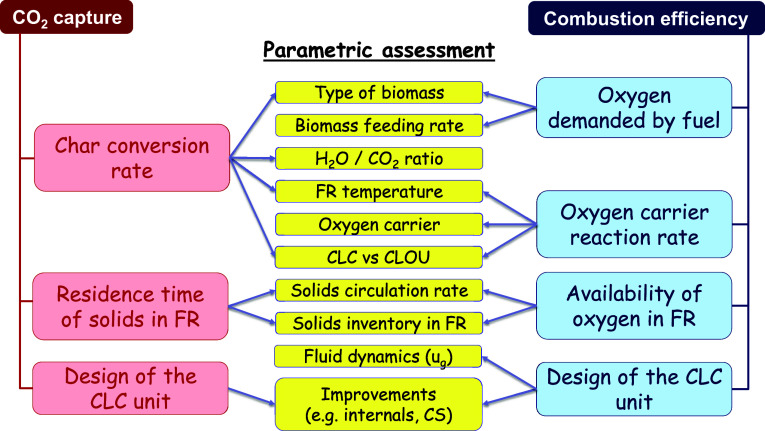
Diagram showing the influence of operating conditions on both the CO_2_ capture rate and combustion efficiency in the Bio-CLC process.

Ω_OD_ is defined as the total oxygen demand of the CLC process, which is related to the required oxygen in the oxygen polishing step. In addition, in the 0.5 kW_th_ CLC unit, the denominator in eq 7 does not include the oxygen to oxidize unconverted solid carbon escaping with gases from the FR.

There is a cross effect between char conversion and combustion efficiency. Thus, the combustion efficiency may decrease when char conversion in the FR increases, since the oxygen carrier will be able to react with a higher amount of fuel in the gaseous phase. To overcome this effect, the combustion efficiency in the FR, η_comb,FR_, and the oxygen demand in the FR, Ω_FR_, are defined considering only the fuel being converted in the FR. A detailed description of the differences between Ω_OD_ and Ω_FR_ is found elsewhere.^28^

8 $$\eta_{comb,FR}=1-\Omega_{FR}=1-\frac{oxygen\;demanded\;\text{for}\;the\;complete\;combustion\;of\;gases\;from\;the\;FR}{oxygen\;demanded\;\text{for}\;complete\;combustion\;of\;fuel\;converted\;in\;the\;FR}$$

### 3.3 Materials

The identification of a suitable material to be used as an oxygen carrier is a keystone for the development of chemical looping technologies. It is desirable for an oxygen carrier material to have a number of characteristics, including favorable thermodynamics, high reactivity, attrition resistance, and proper fluidization with no tendency toward agglomeration. Although abundant and low-cost materials may not meet some of them, such as reactivity and attrition resistance, they are often preferred with biomass in *i*G-CLC. Thus, some chemical interaction between ash and compounds in the oxygen carrier particles is expected, which may be detrimental to the properties sought. This issue may become more relevant than other properties, and it may hinder the use of synthetic materials with better initial properties, in comparison to low-cost materials. Anyway, there are no alternative materials to the synthetic ones for CLOU.

Among different oxygen carriers developed and/or used at ICB-CSIC, several materials have been tested with different solid fuels in the 0.5 and 50 kW_th_ CLC units; see Table 1. In total, about 520 h of combustion has been carried out, being approximately half in the 0.5 kW_th_ CLC unit in CLOU mode. Different oxygen carriers have been used, including natural ores for the *i*G-CLC mode and synthetic materials for CLOU, mostly Cu-based but also one MnFe mixed oxide. Note that the MnFe mixed oxide has a limited oxygen uncoupling capability, requiring the supply of a relevant fraction of lattice oxygen through gas–solid reaction. This mode was also named as chemical looping assisted by oxygen uncoupling.^58^ Table 2 compiles the main properties of these oxygen carrier materials, which are briefly described below:

- Fe-ore Tierga: material with high Fe content (76% Fe_2_O_3_) supplied by PROMINDSA from a hematite mine in Tierga (province of Zaragoza, Spain). Particles were crushed and sieved to +0.1–0.3 mm to be used in the 0.5 kW_th_ unit and +0.1–0.2 mm for the 50 kW_th_ unit. A lower particle size was used in the 50 kW_th_ unit to promote the solid entrainment rate from the FR. Then, particles were calcined at 950 °C for 12 h. This material has high reactivity with H_2_ and CO.^59^
- Mn ores: two Mn-based ores were used as an oxygen carrier for *i*G-CLC with biomass, one from Gabon (67.5% Mn_3_O_4_, 10.8% Fe_2_O_3_) and the other from South Africa (65.6% Mn_3_O_4_, 18.6% Fe_2_O_3_). Particles with +0.1–0.3 mm diameter were obtained after a crushing and sieving process. After the calcination process (800 °C for 2 h), manganese and iron oxides did not form mixed oxides.
- Cu-based oxygen carriers: materials with oxygen uncoupling capability were mainly based on copper oxide. They were prepared by granulating a powder mixture of CuO with other active or inert compounds to obtain the following materials in the +0.1–0.3 mm interval: 60CuO/MgAl_2_O_4_ (60% CuO, spray drying with MgAl_2_O_4_, calcined at 1100 °C for 24 h), 30CuO/MnFe (granulated with Mn_3_O_4_ and Fe_2_O_3_ in a spouted bed, calcined at 1100 °C for 4 h), 30CuO/MnFe/Kao (granulated with Mn_3_O_4_, Fe_2_O_3_ and kaolin in a spouted bed, calcined at 1050 °C for 4 h), and Cu34Mn66 (34% CuO granulated with Mn_3_O_4_ in a spouted bed, calcined at 1125 °C for 2 h). A magnetic MnFe mixed oxide was formed when CuO was granulated with Mn_3_O_4_ and Fe_2_O_3_. This property was desired to separate the oxygen carrier particles from ash by magnetic methods. In the 30CuO/MnFe/Kao material, kaolin was added to improve the mechanical and chemical performance. The Cu34Mn66 material was mainly formed by a CuMn mixed oxide.
- MnFe mixed oxide: the Mn66FeTi7 material was prepared by spray granulation in a spouted bed to obtain particles in the +0.1–0.3 mm interval. After a calcining process (1050 °C for 2 h), the bixbyite (Mn_0.66_Fe_0.34_)_2_O_3_ phase was formed, which has oxygen uncoupling capability when reduced to spinel (Mn_0.66_Fe_0.34_)_3_O_4_. During its use in the CLC unit, limited regeneration to bixbyite was observed in the AR. Thus, supplementary oxygen was supplied by gas–solid reaction taking lattice oxygen in the reduction of spinel to manganowüstite (Mn_0.66_Fe_0.34_)­O. The spinel phase offers magnetic properties, which is useful for the oxygen carrier separation from ash.

These oxygen carriers were used to burn different kinds of biomass in the 0.5 and 50 kW_th_ CLC units. The most used biofuel in the 0.5 kW_th_ plant was pine wood. This biofuel is characterized by a low ash content because only the woody part was processed in its preparation. Olive stones and almond shells were also widely used, as they are agricultural residues with high annual production in Spain. All of them were used in the shape of particles sieved to +0.5–2.0 mm. In addition, a residue from the pig breeding (swine manure) was used as fuel in CLOU. The solid fraction of swine manure was dried and then milled and sieved to +0.5–3.35 mm. This material was characterized by high ash (29%) and nitrogen (2.9%) content. A description of this biofuel can be found elsewhere.^54^ In the 50 kW_th_ plant, olive stone was the biofuel that has been used the most in the *i*G-CLC mode. Other biomasses used in CLOU were pine wood and pine forest residue (PFR). PFR was a pelletized material in the shape of cylinders with a diameter of 6 mm and an average length of 15 mm. The analysis of these biomasses is shown in Table 3.

**Table 3:** Proximate and Ultimate Analysis (wt % as Received) of Biomasses Used in the 0.5 and 50 kW_th_ CLC Units

	pine wood	PFR (pellets)	olive stone	almond shell	swine manure
proximate analysis					
moisture	4.2	3.3	9.4	2.3	2.2
ash	0.4	1.3	0.8	1.1	29.0
volatile matter	81.0	77.2	72.5	76.6	57.8
fixed carbon	14.4	18.3	17.3	20.0	11.0
ultimate analysis					
C	51.3	51.5	46.5	50.2	36.2
H	6.0	5.8	4.8	5.7	4.5
N	0.3	0.3	0.2	0.2	2.9
S	0.0	0.0	0.0	0.0	0.8
O	37.8	37.8	38.3	40.5	24.4
LHV (kJ/kg)	19,140	17,941	16,807	18,071	13,649
Ω_f_ (kg O_2_/kg fuel)/(kg O_2_/MJ)	1.5/0.077	1.5/0.081	1.2/0.074	1.4/0.077	1.1/0.080

## 4 Results: Achievements and Advancements on Bio-CLC

The Combustion and Gasification group at Instituto de Carboquímica (ICB-CSIC) has extensive operational experience in the running of chemical looping units for the combustion (CLC) and gasification (CLG) of solid fuels with CO_2_ capture. Focusing on CLC, Figure 7 graphically shows the experience achieved, with a total of 2018 h of hot operation, of which 1184 h corresponds to combustion with fuel feeding. At the beginning, the combustion of fossil fuels such as anthracite, bituminous coal, and brown coal was evaluated.^21^ Currently in Europe, there is a decrease in interest in the use of fossil fuels and a push toward renewable energy, so the use of biomass is now more interesting. This change in the paradigm in energy production motivates the increase in operational experience with different types of biomasses currently accumulating a total of 517 h of combustion; see Table 1. In addition, the Bio-CLC process can be integrated in the BECCS concept for CDR. One of the main differences between using coal or biomass is the higher volatile content in the latter. This fact makes it difficult to obtain high combustion efficiencies in *i*G-CLC mode, and the CLOU operation is a preferred option with biomass (301 h), as it will be discussed in this work. Below is a detailed analysis of the main results obtained during the operation of the 0.5 and 50 kW_th_ CLC units with biomass, related to CO_2_ capture and combustion efficiency as well as tar and N compounds.

**Figure 7:**
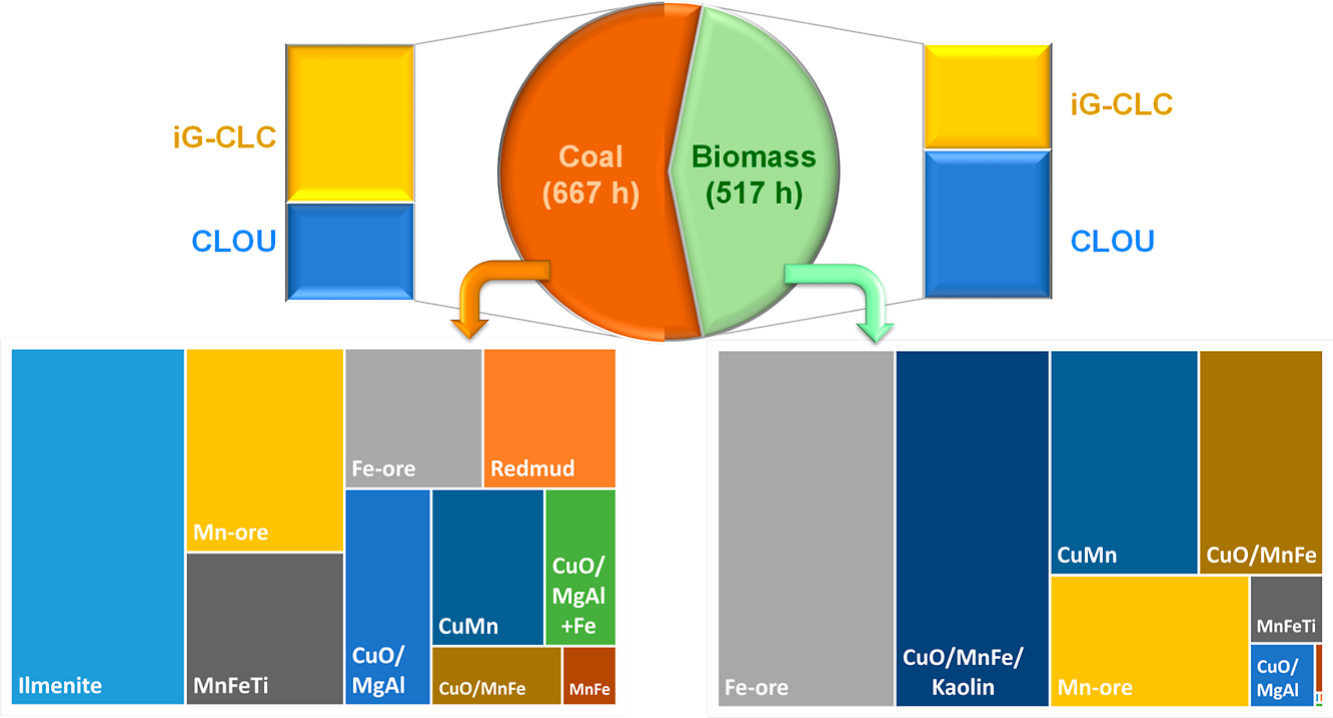
Distribution of operational experience at ICB-CSIC in CLC with solid fuels (coal and biomass), as well as in the use of different oxygen carriers for *i*G-CLC and CLOU modes.

### 4.1 CO_2_ Capture Rate: The Conversion of the Solid Fuel in the FR

The main operating parameters affecting the CO_2_ capture are the temperature and mean residence time of solids in the FR. Figure 8 shows most of the results obtained in the 0.5 kW_th_ CLC unit burning pine wood both in *i*G-CLC and CLOU modes. In both cases, CO_2_ capture values close to 100% are achieved, which is quite different from what happened with coal. Thus, CO_2_ capture values in the 80–90% interval could be achieved in *i*G-CLC mode with lignite, but values lower than 60% were obtained with bituminous coals and anthracite.^21^ Higher values were achieved in CLOU with coal^60^ but always lower than those here showed with biomass. These differences are due to the effect of the solid fuel characteristics on the CO_2_ capture, namely, the composition and the reactivity. Assuming all carbon in volatile matter is evolved in the FR, eq 9 relates the fraction of carbon being fixed in the fuel, *f*_C,fix_, and the char conversion in the FR, *X*_char_, with the CO_2_ capture. Thus, the CO_2_ capture increases as the fixed carbon fraction decreases or char conversion increases. The fraction of carbon being fixed, *f*_C,fix_, is 0.28 for pine wood, 0.37 for olive stone, and 0.40 for almond shell, which are values quite lower than those usually found for coal (*f*_C,fix_ = 0.8–0.95). In addition, the reactivity of char from biomass is higher than the reactivity of char from coal.^43^ This fact is especially relevant when CO_2_ is used as a gasifying agent.

9 $$\eta_{CC}=f_{C,vol}+f_{C,fix}X_{char}=1-\left(1-X_{char}\right)f_{C,fix}$$

**Figure 8:**
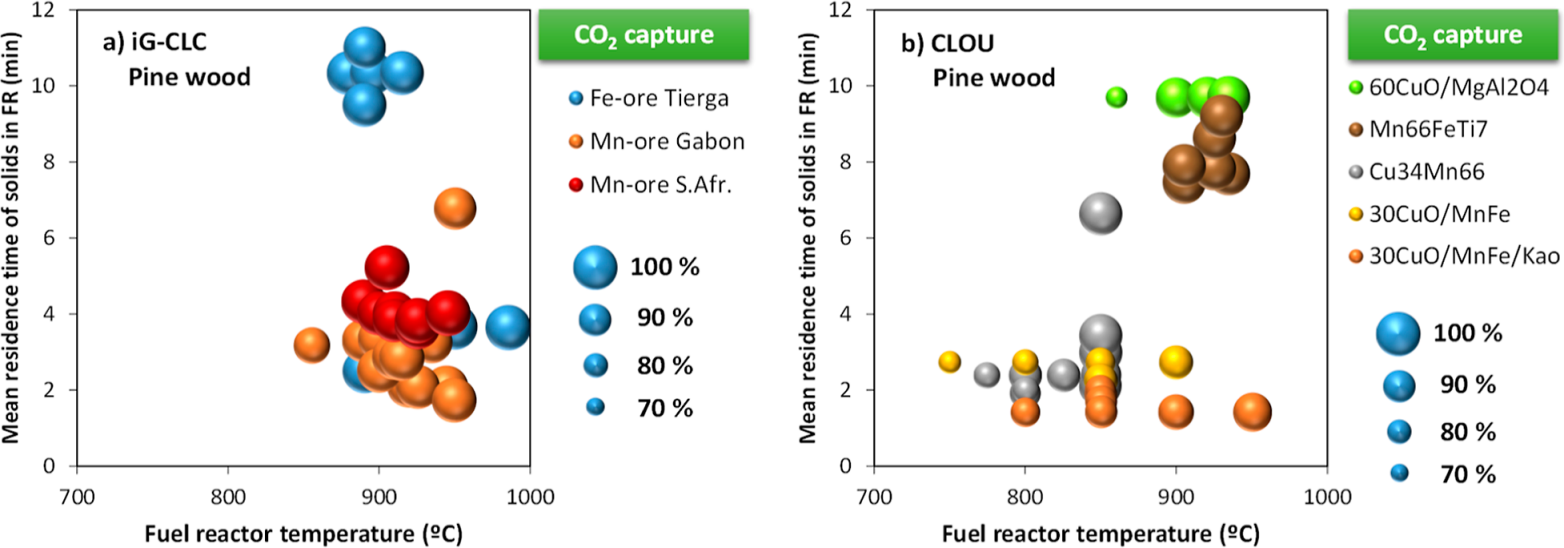
CO_2_ capture achieved in the 0.5 kW_th_ CLC unit as a function of the temperature and the mean residence time of solids in the FR. Fuel: pine wood. Size of the spheres indicates the CO_2_ capture. Data were compiled from the corresponding references in Table 1.

In *i*G-CLC, high CO_2_ capture rates are achieved regardless the oxygen carrier used (e.g., Fe ore or Mn ores) and the fluidizing gas used, namely, H_2_O or CO_2_. In fact, the use of CO_2_ can improve the CO_2_ capture,^48^ contrary to what happened with coal.^61^ The mean residence time of solids in the FR was high enough to allow almost complete char conversion, which is related to the high CO_2_ capture values observed. Unfortunately, the effect of the mean residence time of solids in the FR could not be inferred from tests in the 0.5 kW_th_ CLC unit, and uniquely, the effect of the FR temperature and type of biomass is clearly established. Figure 9a shows that a temperature higher than 900 °C in the FR is required to achieve CO_2_ capture values close to 100% regardless of the oxygen carrier used. In these cases, the mean residence time of solids is about 3–4 min.

**Figure 9:**
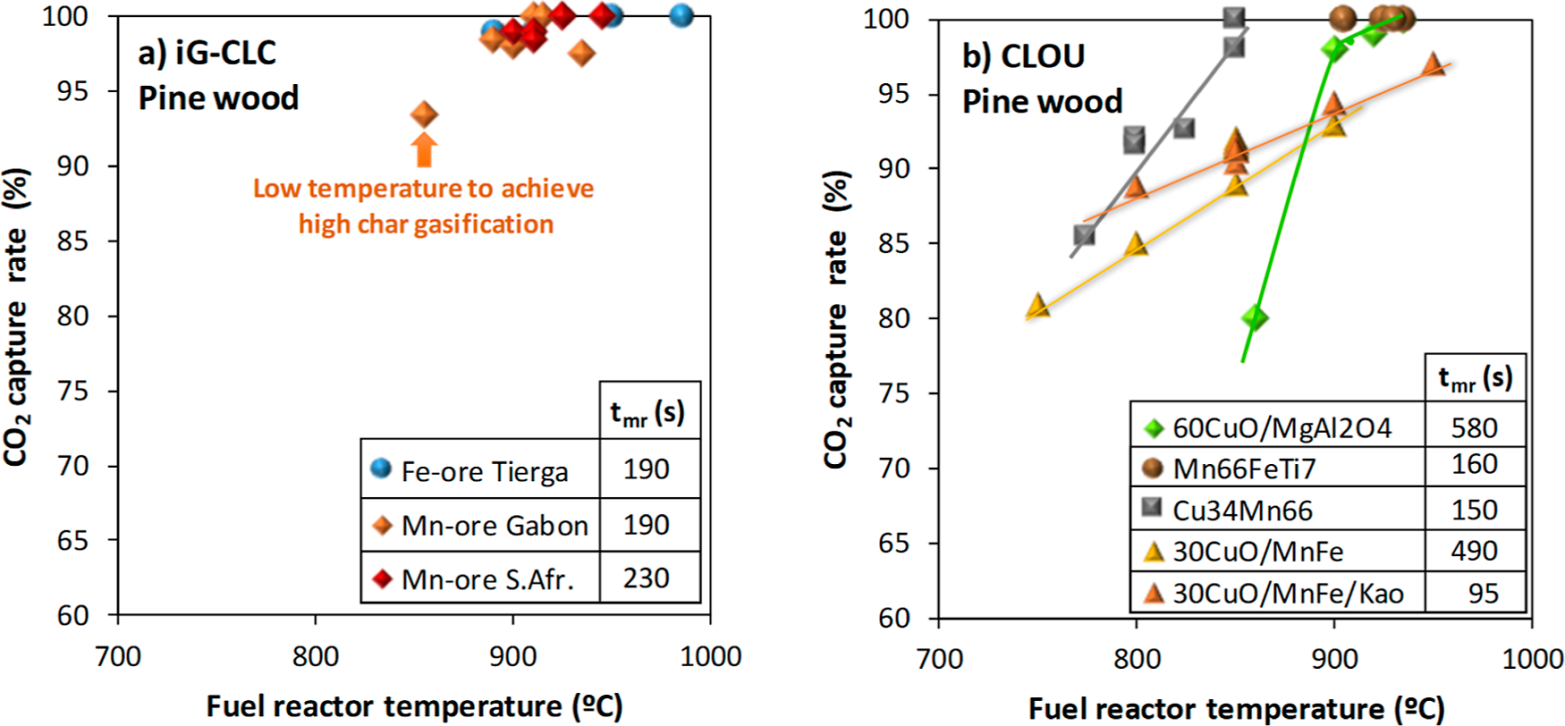
CO_2_ capture as a function of the FR temperature for different oxygen carriers in (a) *i*G-CLC using H_2_O or CO_2_ as a fluidizing gas and (b) CLOU in the 0.5 kW_th_ CLC unit with pine wood as the fuel using N_2_ as a fluidizing gas, except with Mn66FeTi7 when CO_2_ was used instead.

On the contrary, relevant differences on the CO_2_ capture with the operating conditions are observed during CLOU operation; see Figure 8b. Note that in these cases, N_2_ was used as a fluidizing gas in the FR, except for Mn66FeTi7 in which case CO_2_ was used. In the industrial process, CO_2_ or H_2_O would be used as a fluidizing medium. Thus, Figure 9b shows that the CO_2_ capture was close to 100% with Mn66FeTi7 in all tests. Then, the char conversion and the CO_2_ capture were in line to what was observed in the *i*G-CLC mode. However, the relevance of the oxygen uncoupling mechanism on the char conversion could not be evaluated when CO_2_ was used as the fluidizing gas. CO_2_ gasification was the relevant conversion way in *i*G-CLC. But by feeding CO_2_ in CLOU, there is one additional way to convert solid carbon in char, namely, the combustion by released O_2_. The relevance of the oxygen uncoupling to the fuel conversion can be evaluated in greater detail by fluidizing with an inert gas, such as N_2_, without interference of the char gasification with the fluidizing medium. Therefore, most of CLOU tests were performed using this inert gas as a fluidizing medium. But it should be considered that higher CO_2_ capture rates would be expected in an industrial unit where CO_2_ or H_2_O was used as a fluidizing gas.

The effect of the mean residence time of solids in the FR, *τ*_*FR*_, on the CO_2_ capture could not be clearly established due to a lack of information from the tests, but it seems that both FR temperature and the type of the oxygen carrier are primary factors affecting the CO_2_ capture. With all Cu-based materials, the increase in the CO_2_ capture with the FR temperature is expected as the char conversion rate increases with temperature. Also, in general, higher CO_2_ capture rates are achieved in tests with Cu34Mn66 and 30CuO/MnFe/Kao materials, although *τ*_*FR*_ was lower than with 60CuO/MgAl_2_O_4_ or 30CuO/MnFe, revealing the relevance of the oxygen carrier characteristics on the char conversion by oxygen uncoupling. Thus, the temperature to achieve CO_2_ capture values close to 100% is a function of the oxygen carrier used, being about 850 °C for Cu34Mn66 and 920 °C for 60CuO/MgAl_2_O_4_. A thermodynamic analysis of the oxygen uncoupling reaction explains this difference. The active phase in the 60CuO/MgAl_2_O_4_ material is CuO due to low interaction with the MgAl_2_O_4_ support.^62^ At 920 °C, about 2 vol. % O_2_ may be in gases in the FR at equilibrium conditions; see Figure 10. However, the active phase in Cu34Mn66 is Cu_1.5_Mn_1.5_O_4_, 850 °C being the temperature to achieve the same 2 vol. % O_2_ at equilibrium conditions.^49^ With both oxygen carriers, the required temperature in the FR to achieve CO_2_ capture values close to 100% is that corresponding to 2 vol. % O_2_ at equilibrium, which seems to be a suitable value to highly convert the char from pine wood. Both, the char conversion rate and CO_2_ capture decrease for lower temperatures due to a combination of the following reasons: lower oxygen concentration at equilibrium, lower oxygen generation rate by the oxygen carrier, and lower reactivity of char conversion. An intermediate behavior is observed for Cu-based materials supported on MnFe mixed oxides. In these cases, the active phases are a combination of CuO and CuMn, CuFe, and CuMnFe mixed oxides,^63^ which justify that, at low temperature, the CO_2_ capture was higher than that for pure CuO. However, at the highest temperatures, the CO_2_ capture is not as high as for 60CuO/MgAl_2_O_4_ due to a lower reactivity of the material.

**Figure 10:**
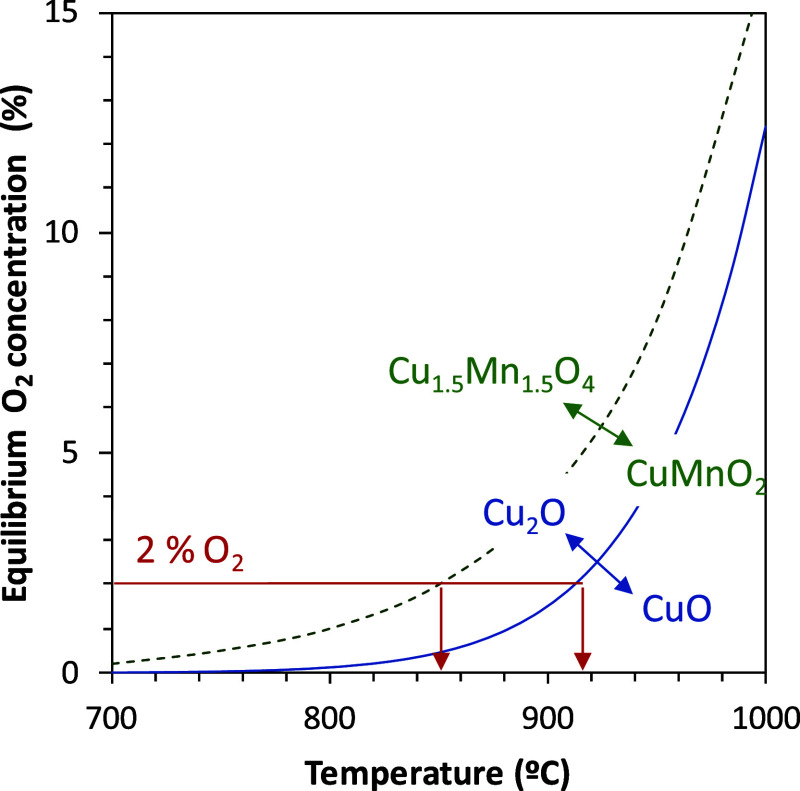
Oxygen concentration at equilibrium conditions for the oxygen uncoupling reactions of CuO and Cu_1.5_Mn_1.5_O_4_, the main active phases in 60CuO/MgAl_2_O_4_ and Cu34Mn66 materials, respectively.

Nevertheless, the use of MnFe mixed oxides as a supporting material is interesting due to its magnetic properties useful for recovering the oxygen carrier from ash particles.^52^ In addition, the actual CO_2_ capture in a CLC unit with CLOU materials is expected to be high due to the fact that values presented here would be improved by the existence of the parallel gasification char reaction by H_2_O or CO_2_ used as a fluidizing gas.

Figure 11 compiles relevant results in *i*G-CLC and CLOU to evaluate the CO_2_ capture as a function of the oxygen carrier or the biomass. Thus, four different biomasses, namely, pine wood, olive stone, almond shell, and swine manure, have been used in the 0.5 kW_th_ CLC unit. The high CO_2_ capture values that can be achieved with all biomasses are remarkable, but some relevant differences are observed under conditions with low char conversion rates, i.e., low temperature and mainly in CLOU mode. The CO_2_ capture decreased following the sequence pine wood > swine manure > almond shell > olive stone. When the char conversion rate was calculated, it was observed that the pine wood was converted faster than almond shell and olive stone.^55^ Differences between almond shell and olive stone could be caused by different τ_FR_ values, which were higher for the almond shell. Anyway, in any case, high char conversion values and CO_2_ capture rates would be expected in a CLC unit, both in *i*G-CLC and CLOU modes, with biomass, and the presence of a CS to improve the CO_2_ capture would not be required, as it was in the case of burning coal.^37^

**Figure 11:**
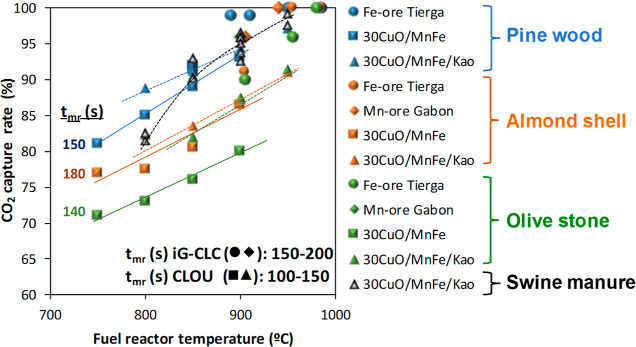
CO_2_ capture in *i*G-CLC and CLOU modes achieved in the 0.5 kW_th_ CLC unit as a function of temperature for several oxygen carriers and biomasses.

High CO_2_ capture rates are also achieved at the 20 kW_th_ scale (in the 50 kW_th_ unit), although the residence time was shorter than in the 0.5 kW_th_ unit; see Figure 12a. This difference is relevant, and it is caused by the different fluidization regime, which is bubbling in the 0.5 kW_th_ unit and turbulent/fast fluidization in the 50 kW_th_ unit. The higher gas velocity in the FR makes that the specific cross section and the solid inventory per thermal power was lower in the 50 kW_th_ unit,^64^ decreasing the mean residence time of solids in the FR for the same specific solid circulation rate.

**Figure 12:**
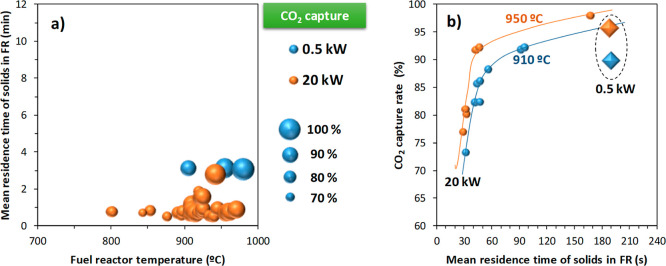
Comparison of the CO_2_ capture achieved at the 0.5 and 20 kW_th_ scales in both CLC units with Fe-ore Tierga and olive stone. Data compiled from the corresponding references in Table 1.

Similar to the results for the 0.5 kW_th_ unit, it is inferred that an increase in the FR temperature improves the CO_2_ capture. In addition, the effect of the mean residence time, τ_FR_, on the CO_2_ capture could be established from results at the 20 kW_th_ scale; see Figure 12b. Thus, the CO_2_ capture greatly improves when τ_FR_ increases up to 1 min, and then, this increase is of lower relevance. From these results, it may be deduced that the CO_2_ capture in the 50 kW_th_ CLC unit was somewhat higher than in the 0.5 kW_th_ unit under similar operating conditions. This effect may be caused by the presence of a CS in the 50 kW_th_ unit. The CS was designed to separate fine coal particles (+0.1–0.2 mm), but it may be able to separate a fraction of unconverted char particles with the size used (+0.5–2.0 mm) to recirculate them to the FR,^38^ increasing in this way the char conversion in the FR. Nevertheless, lower differences among the CLC units are observed if the effect of the CS is discounted.

Among the parameters affecting the char reactivity and the CO_2_ capture shown in Figure 6, the type of the biomass is a key factor to be considered for a proper design of the CLC unit (e.g., residence time of solids in the FR) or for the selection of suitable operating conditions (e.g., FR temperature) in order to achieve high CO_2_ capture rates. Nevertheless, the use of either H_2_O or CO_2_ as a fluidizing medium both in *i*G-CLC or CLOU is of lower relevance, as well as the need for a CS. CO_2_ capture rates in the 90–95% interval are achievable in a common design for circulating fluidized beds with olive stones, and higher values would be expected with the more reactive pine wood. In addition, recent results in CLOU mode indicate that CO_2_ capture values as high as 99% can be obtained at 900 °C with pine wood and the 30CuO/MnFe oxygen carrier in the 50 kW_th_ unit.^36^

### 4.2 Combustion Efficiency: The Fuel Oxidation in the FR

The combustion of a solid fuel in the FR is carried out using oxygen transported from the AR by the oxygen carrier. The direct solid–solid reaction between oxygen carrier particles and the solid fuel has low relevance in the oxygen transfer mechanism in a fluidized bed.^65^ Therefore, the main mechanisms to oxidize the fuel include intermediate stages such as the fuel gasification or the oxygen uncoupling of the oxygen carrier. The efficiency transferring oxygen from the oxygen carrier to the fuel depends on the main mechanisms involved in this process, being more effective the pathway including the oxygen uncoupling.^66,67^ As a consequence, it would be expected that the combustion efficiency in CLOU was higher than that in *i*G-CLC. This fact is confirmed during the combustion of biomass in the 0.5 kW_th_ unit with different oxygen carriers; see Figure 13. Here, the combustion efficiency is evaluated through the total oxygen demand parameter, Ω_OD_, defined by eq 7. This parameter is widely used in the CLC field because it represents the fraction of oxygen required in an eventual oxygen polishing step to achieve the complete combustion of the fuel—see Figure 2—compared to the oxygen required in the alternative oxy-fuel combustion process with CO_2_ capture.

**Figure 13:**
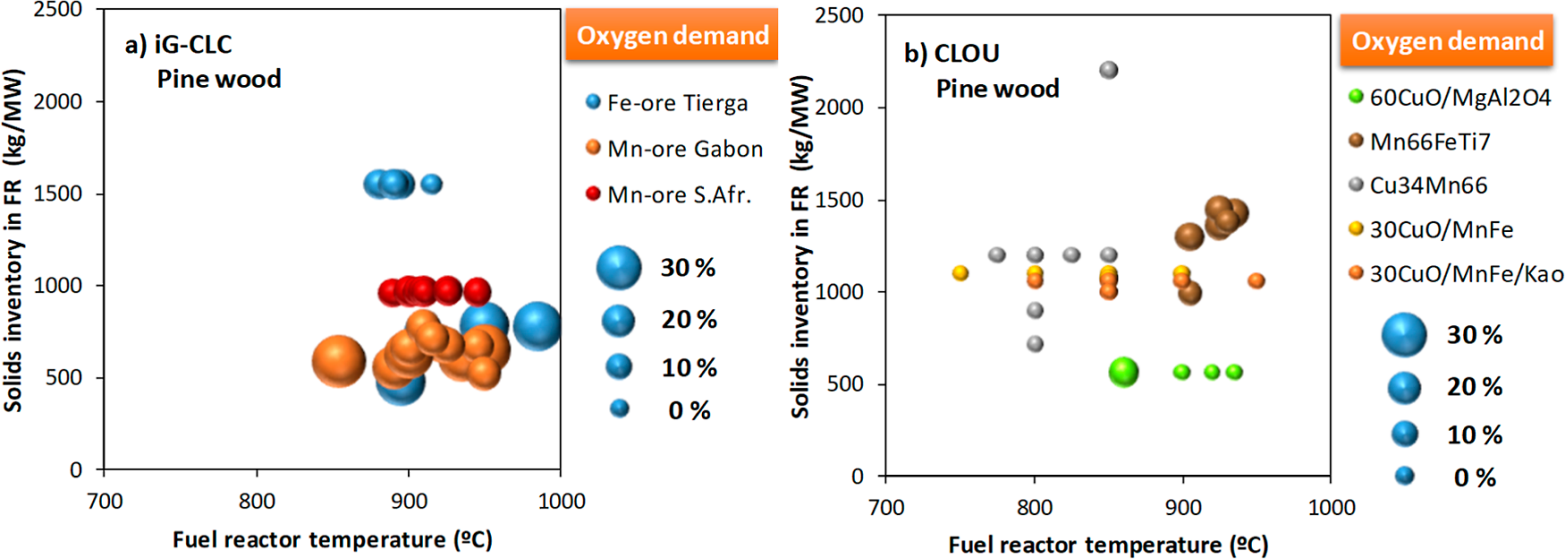
Total oxygen demand, Ω_OD_, as a function of the FR temperature and the solids inventory in the FR in the 0.5 kW_th_ CLC unit with pine wood and different oxygen carriers: (a) *i*G-CLC; (b) CLOU. Data compiled from the corresponding references in Table 1.

In general, complete combustion is achieved in the CLOU process with Cu-based oxygen carriers; see Figure 13b. However, care must be taken to operate at an adequate temperature for sufficient oxygen generation. Thus, temperatures above 900 °C would be required for 60CuO/MgAl_2_O_4_, which is in agreement with what was also observed in the analysis of the CO_2_ capture; see Figure 9b. But with the oxygen carrier based on a MnFe mixed oxide (Mn66FeTi7), the oxygen demand was in the Ω_OD_ = 10–16% interval. This material has oxygen uncoupling capability if its higher oxidation state, namely, bixbyite phase (Mn_0.5_Fe_0.5_)_2_O_3_, is formed in the AR, which is favored as temperature decreases and oxygen concentration increases.^58^ An optimum temperature in the AR of 880 °C was found to maintain a high oxidation rate and to support a temperature higher than 900 °C in the FR, which is required for both fast oxygen uncoupling reaction and the proper conversion of the solid fuel.^68^ Taking this into account, the 0.5 kW_th_ CLC unit was operated with temperatures in the AR and FR of 880 and 925 °C, respectively. Then, the oxygen uncoupling mechanism was promoted by increasing the air excess ratio in the AR from 1.5 to 3.0, which resulted in a decrease in the oxygen demand from 16% to 10%.^56^ Anyway, complete combustion is not achieved due to the limited capability of the MnFe mixed oxide to transfer oxygen via oxygen uncoupling. Then, a relevant amount of oxygen should be transferred via gas–solid reaction of the spinel phase—(Mn_0.5_Fe_0.5_)_3_O_4_—with gaseous products in *i*G-CLC mode.

In the *i*G-CLC process, complete combustion was never achieved; see Figure 13a. Typical values of the total oxygen demand are in the 15–30% interval, and from results in this figure, it is deduced that it decreases with the solid inventory. The effect of the FR temperature seems to be of lower relevance, although it will be discussed in detail below. More than 50% of oxygen demand is due to the presence of CH_4_, which is a typical product in volatile matter, followed by CO and H_2_.^48^ Thus, it is believed that the fuel conversion may be limited by a deficient contact in the bubbling fluidized bed between the oxygen carrier particles in the emulsion phase and volatiles evolved in the plumes inside the bed. The oxygen demand could be decreased to Ω_OD_ ≈ 3% with the Fe-ore Tierga by a combination of high temperature and very high solid inventory. These values are lower than those registered for coal combustion, which usually are in the 3–10% interval,^21^ and it could be as low as Ω_OD_ = 2% with the Fe-ore Tierga.^42^

The relatively low oxygen demand found for the South-African Mn ore, Ω_OD_ ≈ 10%, is relevant, which was due to the higher reactivity of this material, mainly with CO and H_2_.^69^ Some authors found oxygen uncoupling capability for Mn ores,^70^ which may justify the improved combustion of the fuel in tests with solid fuels.^71^ This effect may be related to the high Fe content in these ores which may facilitate the formation of MnFe mixed oxides.^72^ However, the formation of MnFe mixed oxides and the oxygen uncoupling capability was not observed in the Mn ores from South Africa and Gabon tested in CSIC.^69,73^

There are three operational parameters mainly affecting the oxygen demand with solid fuels, namely, the FR temperature, the solid inventory in the FR, and the solid circulation rate in the CLC unit; see Figure 6. Figure 14a shows the oxygen demand with Fe-ore Tierga for different biomasses as a function of the FR temperature. The effect of the solid inventory in the FR on the oxygen demand can be clearly observed for pine wood, decreasing the oxygen demand from 28 to 12% when the solid inventory increased from 475 to 1550 kg/MW_th_. In addition, the type of biomass roughly affects the oxygen demand due to all biomasses having high volatile content, where most of the unburned compounds come from. Oddly, oxygen demand increases with the FR temperature. It would be expected that an increase in the temperature could improve the combustion efficiency due to the corresponding increase in the oxygen transference rate from the oxygen carrier. However, it is necessary to take into account the side effects of the temperature on char conversion. Thus, the char conversion increases with temperature, which is related to the increase in the CO_2_ capture shown in Figure 11. This means that the effective fuel load of the CLC unit increases with temperature because more gasified char demands more oxygen from the oxygen carrier. To overcome this issue, the combustion efficiency in the FR defined by eq 8 may be used to evaluate the FR performance; see Figure 14b. Thus, when the effect of the different fuel amounts to be oxidized is eliminated, the combustion efficiency barely varies with the FR temperature or the type of biomass.

**Figure 14:**
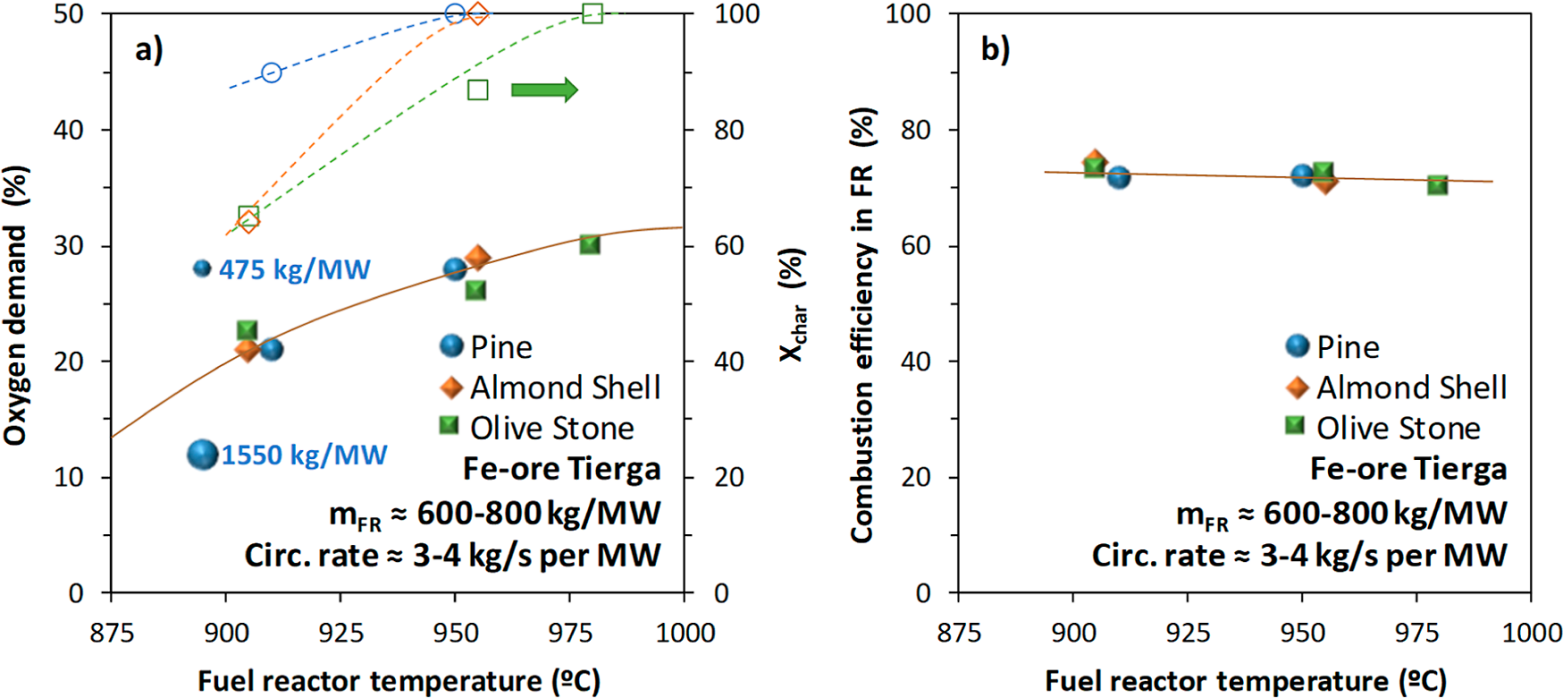
(a) Total oxygen demand, Ω_OD_, and (b) combustion efficiency in the FR, η_comb,FR_, as a function of the FR temperature for different biomasses with Fe-ore Tierga in the 0.5 kW_th_ CLC unit.

The effect of the FR temperature on the oxygen demand may be also evaluated by comparing results for the same value of the char conversion. Available data for that are for the combustion of pine wood with Mn ores; see Figure 15. In this case, the oxygen demand decreases when the FR temperature increases. In addition, the oxygen demand decreases as solids circulation increases. Although the supplied and demanded amount of oxygen is the same, an increase in the solid circulation rate increases the average reactivity of solids in the FR, which may improve the fuel oxidation.^74^ Due to the different solid inventory in the FR for the Mn ore from either Gabon or South Africa, it is not possible to infer the effect of the oxygen carrier reactivity on the oxygen demand at this moment. A discussion on this issue is presented below.

**Figure 15:**
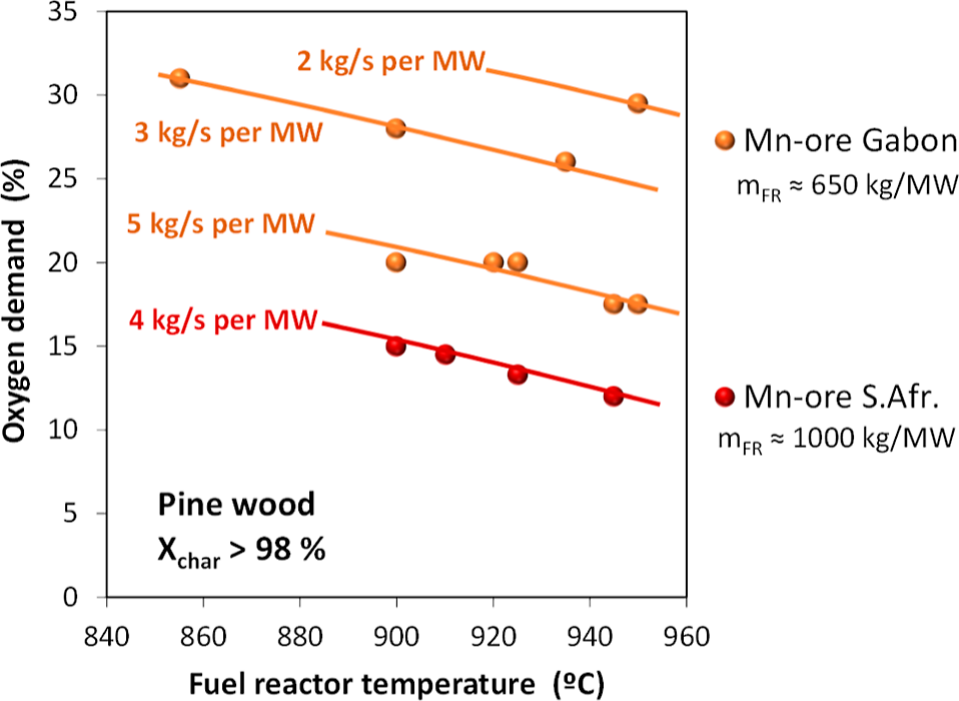
Total oxygen demand, Ω_OD_, as a function of the FR temperature for pine wood with Mn ores in the 0.5 kW_th_ CLC unit for several solid circulation rates.

To make an adequate comparison of the behavior of different oxygen carriers, the results obtained with the same operating conditions must be selected, namely, the same fuel and similar FR temperature and specific solids circulation rate and solids inventory in the FR. Among results available from the operational experience in Table 1, the tests shown in Figure 16 with pine wood are selected to evaluate the combustion performance of biomass depending on the used oxygen carrier. As shown above, complete combustion is achieved with Cu-based materials in CLOU mode. Note that this is achieved with lower FR temperature, solids circulation, or solids inventory than with materials used in *i*G-CLC. Thus, complete combustion was achieved at 800 °C with Cu34Mn66, while 900 °C was required for 60CuO/MgAl_2_O_4_. This difference is due to the temperature required to show that the oxygen uncoupling capability for Cu34Mn66 is lower than for 60CuO/MgAl_2_O_4_; see Figure 10.

**Figure 16:**
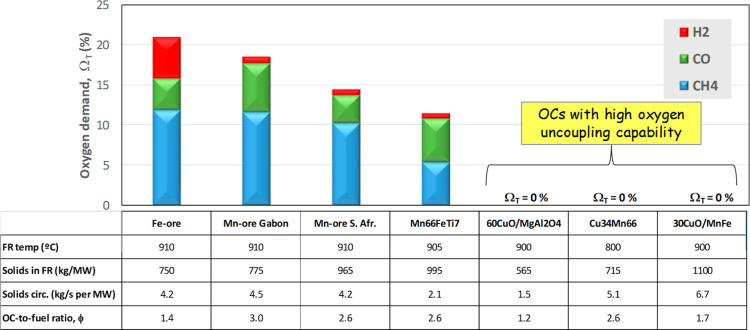
Total oxygen demand for different oxygen carriers both in *i*G-CLC and CLOU mode in the 0.5 kW_th_ CLC unit.

Regarding the natural ores, lower oxygen demand values are obtained with Mn ores compared with the Fe-ore Tierga. This fact is due to the higher reactivity of the Mn ores with the three main gases evolved in the FR, namely, H_2_, CO, and CH_4_. Table 4 shows the rate index as a parameter indicative of the reactivity of an oxygen carrier, which was defined by Johansson et al.^75^ In addition, the average reactivity of the oxygen carrier particles in the FR is a function of the oxygen carrier to fuel ratio, ϕ, defined by eq 10.^74^ Thus, the ϕ parameter is often used to evaluate the performance of a specific oxygen carrier on the combustion efficiency, which depends on the oxygen transport capacity, *R*_OC_.^19^ Usually, the combustion efficiency increases with the ϕ parameter due to the related increase of the solids circulation rate. However, for a proper comparison among different oxygen carriers, the same specific solids circulation rate must be used. Otherwise, different operating conditions—which is defined by the solids circulation rate—would be evaluated by comparing results with the same ϕ value for two oxygen carriers with different *R*_OC_ values. So, results in Figure 16 for Fe and Mn ores are selected having the same specific solids circulation rate; but the ϕ value for the Fe ore is lower than for Mn ores due to its lower *R*_OC_ value.

10 $$\Phi =R_{OC}\frac{{ṁ}_{OC}}{\Omega_{f}{ṁ}_{f}}=(actual\;solid\;circulation\;rate)/(stoichiometric\;flow\;of\;oxygen\;carrier\;to\;supply\;oxygen\;required\;to\;oxidize\;fuel\;to\;{CO}_{2}\text{and}\;H_{2}O)$$

**Table 4:** Oxygen Transport Capacity (*R*_OC_), Stoichiometric Flow of Solids (*ṁ*_OC,st_), and Rate Index for the Oxygen Carrier Particles Used with Biomass in the 0.5 kW_th_ CLC Unit

	*R*_OC_ (%)	*ṁ*_OC,st_ (kg/sMW)	rate index (%/min)		
			H_2_	CO	CH_4_
Fe-ore Tierga	2.5	3.1	12.4	3.4	3.3
Mn-ore Gabon	5.1	1.5	19.2	6.4	9.2
Mn-ore S.Afr.	4.7	1.6	14.2	5.1	5.0
Mn66FeTi7	9.5	0.8	8.5	2.0	4.5
60CuO/MgAl_2_O_4_	6.0	1.3	n.a.	n.a.	n.a.
Cu34Mn66	4.0	1.9	n.a.	n.a.	n.a.
30CuO/MnFe	2.0	4.0	n.a.	n.a.	n.a.

When both Mn ores are compared, lower oxygen demand values are obtained with the South African one. However, it is observed that its reactivity is lower than that of the Mn ore from Gabon. It may be deduced that the lower oxygen demand with the Mn ore from South Africa is due to the higher solid inventory in the FR, which is a relevant parameter affecting the oxygen demand in the 0.5 kW_th_ CLC unit. Unfortunately, there are no results available with more similar conditions. The improvement in the combustion degree is due to the lower fraction of H_2_ in the combustion gases, which is the gas with the highest reactivity with the oxygen carriers. More than half of the oxygen demand is due to CH_4_, which is the gas with the lower reactivity as well as a characteristic gas in volatile matter. The effect of poor contact between volatiles and oxygen carrier particles on the presence of unburnt products was already discussed above.

Interestingly, the CH_4_ fraction is reduced when the Mn66FeTi7 material is used, although its reactivity is lower than that for Mn ores; see Table 4. The oxygen uncoupling capability of the MnFe mixed oxide in Mn66FeTi7 is relevant to decrease the oxygen demand burning biomass, but it is limited to achieve complete combustion, as is achieved with the Cu-based materials.

In addition to results obtained in the 0.5 kW_th_ CLC unit, olive stone was also burnt at the 20 kW_th_ scale with Fe-ore Tierga, i.e., *i*G-CLC mode.^57^ Figure 17a shows a general overview of the oxygen demand obtained as a function of the solids circulation rate and the solids inventory in the FR. These results were obtained in a temperature interval of 850–960 °C. In general, the oxygen demand decreases as both circulation and inventory of solids in the FR increase. After a comprehensive analysis of the results, it is concluded that one of the main factors affecting the oxygen demand is the char conversion, as shown in Figure 17b. Thus, the oxygen demand increases with the char conversion because the amount of fuel demanding oxygen from the oxygen carrier—i.e., reducing gases such as H_2_ and CO—increases. Thus, oxygen demand values as low as Ω_OD_ = 5% may be achieved but at the expense of having low CO_2_ capture rates. If a minimum CO_2_ capture of 90% is fixed, in the best of cases, an oxygen demand of Ω_OD_ = 12% is estimated.

**Figure 17:**
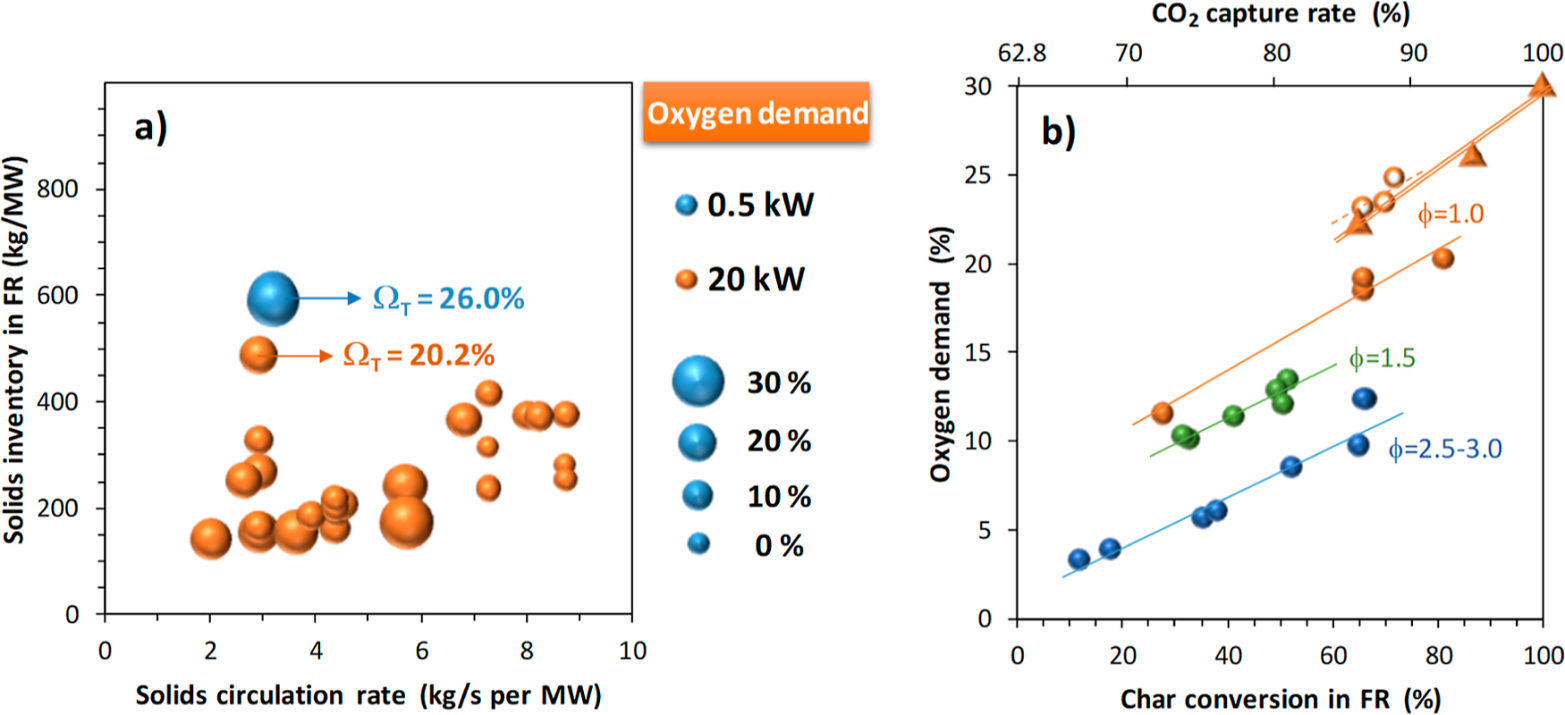
Total oxygen demand, Ω_OD_, in the 50 kW_th_ CLC unit with Fe-ore Tierga and olive stone as a function of the (a) circulation rate and the solids inventory in the FR and (b) char conversion in the FR for different oxygen carrier to fuel ratios (ϕ), solids inventory in the FR (*m*_FR_), and FR temperature (*T*_FR_). Symbols in (b): ○ (orange) (*m*_*OC*_ = 3 kg/s per MW_th_; *m*_FR_ = 150 kg/MW_th_; *T*_FR_ = 910–920 °C); ● (orange) (*m*_OC_ = 3 kg/s per MW_th_; *m*_FR_ = 250–500 kg/MW_th_; *T*_FR_ = 920–940 °C); ● (green) (*m*_OC_ = 4.4 kg/s per MW_th_; *m*_FR_ = 200 kg/MW_th_; *T*_FR_ = 920–940 °C); ● (blue) (*m*_OC_ = 7.2–8.8 kg/s per MW_th_; *m*_FR_ = 200–400 kg/MW_th_; *T*_FR_ = 850–960 °C). For comparison purposes, results in the 0.5 kW_th_ CLC unit are also shown: ▲ (orange) (*m*_OC_ = 3 kg/s per MW_th_; *m*_FR_ = 600 kg/MW_th_; *T*_FR_ = 900–980 °C).

Results in Figure 17b are ordered according to the oxygen carrier to fuel ratio (ϕ). Thus, the oxygen demand decreases with the increase of the ϕ parameter, which is related to an increase in the solids circulation rate. However, increasing the char conversion with high ϕ values requires the increase of the solids inventory in the FR to avoid the decrease of the mean residence time of solids, which could decrease the char conversion; see Figure 12. Both the FR temperature and the solids inventory in the FR have low relevance beyond the side effect it has on the char conversion; see Figure 12. Only in the case that solids inventory values in the FR are as low as 150 kg/MW_th_ is an increase in the oxygen demand observed. Increasing the solids inventory above 200 kg/MW_th_ has low relevance due to the fact that most of the solids are accumulated in the dense bed, which is not very effective in burning gasification gases.

From the results in Figure 17a, it can be intuited that the oxygen demand in the 50 kW_th_ unit is lower than that in the 0.5 kW_th_ unit. Note that, in general, the specific solids inventory in the FR is higher in the 0.5 kW_th_ unit due to its design as a bubbling fluidized bed with the exit of solids by overflowing toward the AR. However, the comparative analysis should be completed by considering the effect of the char conversion on the oxygen demand. Thus, Figure 17b shows that for the oxygen carrier to fuel ratio of ϕ = 1, similar oxygen demand values are achieved with 600 kg/MW_th_ in the 0.5 kW_th_ unit and 150 kg/MW_th_ in the 50 kW_th_ unit. In fact, the oxygen demand in the 50 kW_th_ unit could be decreased by increasing the solids inventory to 250 kg/MW_th_. Therefore, it may be concluded that the fluid dynamics of the FR in the 50 kW_th_ unit—fast fluidization with a relevant dilute phase—improved the gas–solid contact and, eventually, a better oxidation of volatiles is achieved.^21,64^ In fact, the fraction of CH_4_ in the combustion gases from the 50 kW_th_ unit was lower than that from the 0.5 kW_th_.

Recently, some CLOU tests have been carried out with the 30CuO/MnFe material burning pine wood, PFR (pellets), and olive stones. For comparison purposes with results showed in this review, a selection of the obtained results is presented in Table 5. Contrary to what was observed in the 0.5 kW_th_ CLC unit, complete combustion was never achieved. Combustion of PFR showed a relatively low oxygen demand of Ω_OD_ = 2.1%. This fuel was pelletized, and then, a more homogeneous pyrolysis would be expected, which may improve the gas–solid contact and the O_2_–fuel mixture in CLOU mode. But higher oxygen demand values were obtained for pine wood and especially for olive stones.

**Table 5:** Main Operating Conditions and KPIs for CLOU Operation in the 50 kW_th_ Unit with 30CuO/MnFe as the Oxygen Carrier

	pine wood	PFR (pellet)	olive stones
FR temperature (°C)	881	875	880
solids inventory in FR (kg/MW_th_)	220	165	260
solids circulation (kg/s per MW_th_)	6.2	6.6	7.2
mean residence time of solids, τ_char_ (s)	35	25	36
char conversion in FR, *X*_char_ (%)	83	77	54
CO_2_ capture, η_CC_ (%)	97	95	86
oxygen demand, Ω_OD_ (%)	5.6	2.1	7.1

Note that H_2_O and/or CO_2_ was used as the fluidizing gas in the 50 kW_th_ unit, while inert N_2_ was used in the 0.5 kW_th_ unit. Likely, char gasification in the upper part of the reactor releases some H_2_ and CO that could not be oxidized due to the low concentration of solids in the exit zone. This effect may be more relevant, as the char conversion is lower due to a higher char concentration in the dilute region. This may be the case for olive stones, which showed the highest oxygen demand among the used biofuels.

When compared to the *i*G-CLC operation with Fe-ore Tierga under similar operating conditions, the CO_2_ capture in CLOU at 880 °C with olive stones was similar to that achieved in *i*G-CLC at 950 °C and higher than that at 910 °C (η_CC_ ≈ 77%); see values at τ_char_ = 36 s in Figure 12b. In addition, similar oxygen demand values are achieved in both *i*G-CLC and CLOU modes with olive stones; see values at ϕ = 2.5–3 for *X*_char_ = 54% in Figure 17b. The temperature in CLOU mode was somewhat lower than that considered optimal in operation with the 30CuO/MnFe material, namely, 900–950 °C. Figure 18 shows that the oxygen demand monotonically increases with the CO_2_ capture because more gasification products are generated, i.e., the oxygen carrier has to supply more oxygen in *i*G-CLC mode. The same trend is observed in CLOU at low temperatures. But the oxygen demand decreased at higher temperatures, which reveals that the oxygen uncoupling mechanism becomes relevant at temperatures above 850 °C. Unfortunately, it was not possible to increase the temperature above 890 °C in the 50 kW_th_ unit due to heat losses related to the high solids circulation rate required. But an improvement in the CLOU performance would be expected by increasing the FR temperature in CLOU mode.

**Figure 18:**
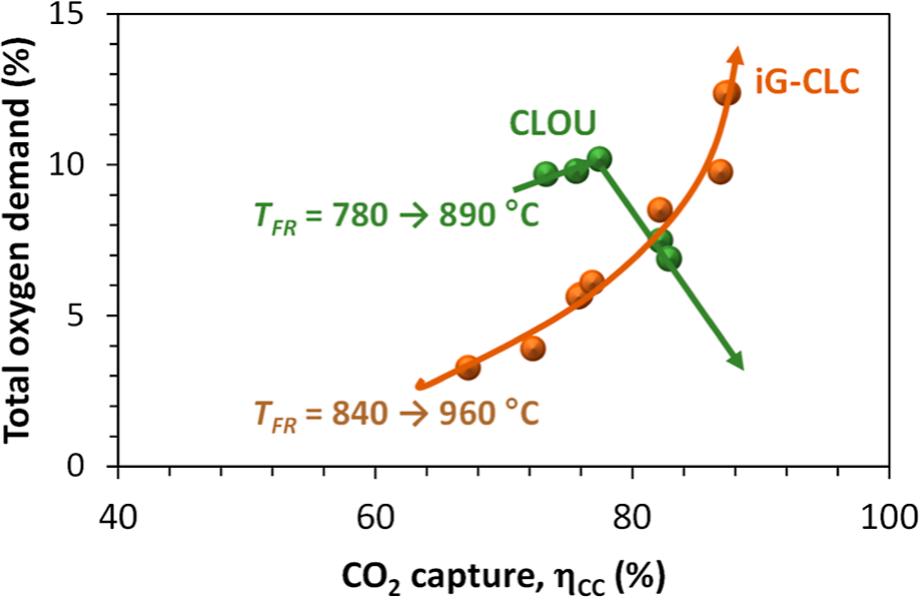
Total oxygen demand, Ω_OD_, as a function of the CO_2_ capture, η_CC_, burning olive stone in the 50 kW_th_ CLC unit in *i*G-CLC with Fe-ore Tierga and CLOU with 30CuO/MnFe. Operating conditions in *i*G-CLC:^57^ *m*_OC_ = 7–8 kg/s per MW_th_; *m*_FR_ = 250–400 kg/MW_th_; τ_FR_ = 30–55 s; in CLOU:^36^ *m*_OC_ = 6 kg/s per MW_th_; *m*_FR_ = 250 kg/MW_th_; τ_FR_ = 40 s.

### 4.3 Tar Compounds

Among unconverted products in the FR, H_2_, CO, and CH_4_, as well as other light hydrocarbons, are considered the majority and have a greater influence on the oxygen demand. In addition, a significant amount of tar is produced during pyrolysis of biomass. The existence of unconverted tar evolved during the biomass conversion may cause operating problems downstream the FR in the CCS chain, namely, the compression, transport, and storage steps. To prevent possible problems, it is necessary to identify and quantify the possible tar compounds in the combustion gases.

Figure 19 compiles the tar content identified in campaigns with different oxygen carriers in pine wood. In addition, an exhaustive evaluation of the tar compounds has been done with Fe-ore Tierga with different biomasses.^46^ In general, the tar concentration was in the ranges of 2.5–4.3, 3.5–3.7, and 2.6–3.0 g/m^3^ (STP) with pine wood, olive stones, and almond shell, respectively. Thus, the highest amount of tar in the CO_2_ stream—4.3 g/m^3^ (STP)—is observed with Fe-ore Tierga and pine wood, which is related to the lower combustion efficiency achieved among the tested oxygen carriers; see Figure 16. A minimum value of tar with pine wood of 0.2 g/m^3^ (STP) was obtained with Fe-ore Tierga with high solid inventory in the FR which promoted the oxidation of all gases.^45^ With the Mn ore from Gabon, the tar content was in the 1.9–3.2 g/m^3^ (STP) interval. A value as low as 0.3 g/m^3^ (STP) was found with Mn ore S. Africa^48^ and somewhat less −0.2 g/m^3^ (STP)—with Mn66FeTi7. Naphthalene is found as the main compound in all cases, with phenanthrene and acenaphthylene as other relevant compounds. Also, the presence of styrene should be mentioned when pine wood was burned with Fe-ore Tierga.

**Figure 19:**
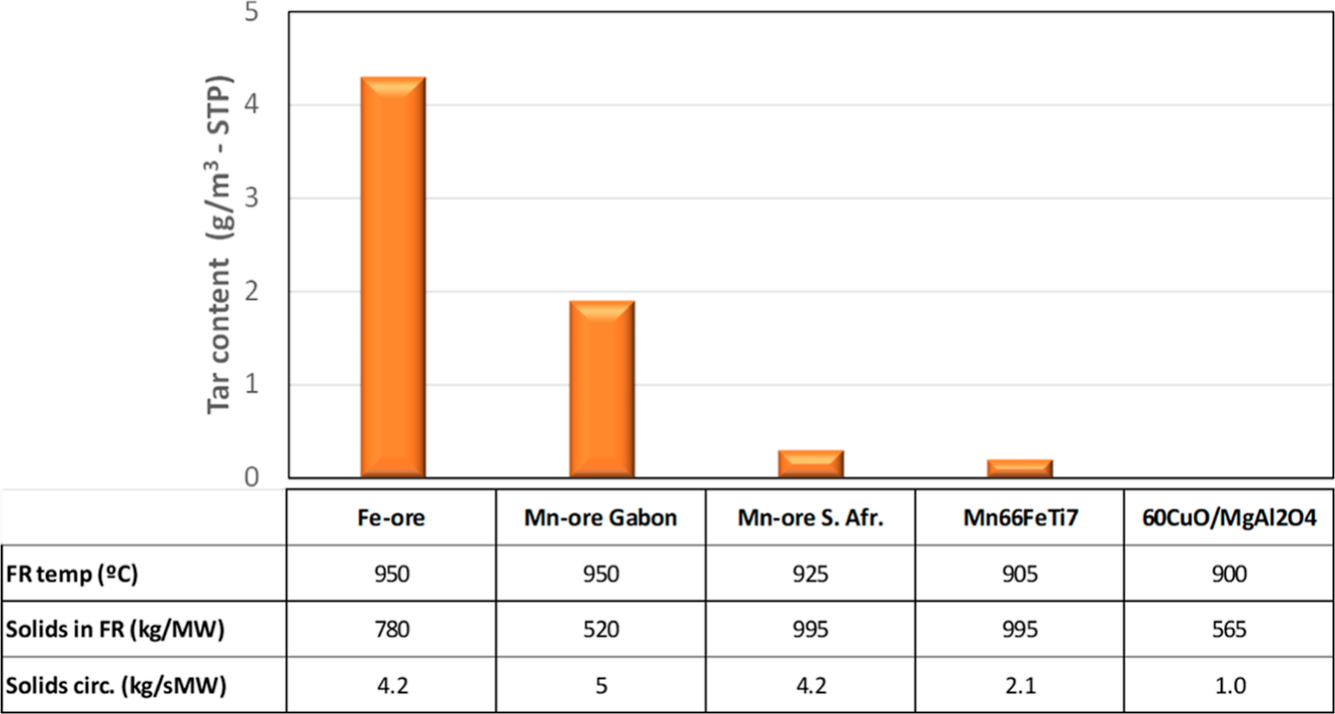
Tar concentration in the CO_2_ stream from the FR with pine wood and different oxygen carriers in the 0.5 kW_th_ CLC unit.

Even in the cases with a lower presence of tar in the CO_2_ stream, some fouling and blocking problems could still be expected. Nevertheless, these amounts would be highly reduced if an oxygen polishing step is implemented in order to oxidize unburnt products from the FR. Note that tar compounds may demand up to 1% of the total oxygen required to burn the fuel, which should be considered to design the oxygen polishing step.

In CLOU, no tar is detected when complete combustion is achieved in the 0.5 kW_th_ unit, which has been evaluated for 60CuO/MgAl_2_O_4_ with pine wood, olive stones, and almond shell and 30CuO/MnFe/Kao with burning swine manure.

### 4.4 Fate of Fuel-N

In a CLC process, thermal NOx not generated due to combustion is carried out at a relatively low temperature and without flame.^76^ However, nitrogen in biomass can be released in the FR and be oxidized. Nitrogen emitted from the FR affect the CO_2_ purity and quality.^77,78^ In addition, nitrogen in char particles may reach the AR and be released in the exhaust air, which should fulfill the legal emission limit to the atmosphere, which is 200 mg/m^3^ (STP) for NOx in EU.^79^ To properly evaluate the N speciation in the FR (e.g., to know if fuel-N is emitted as NOx or N_2_), tests should be done in the absence of externally supplied gaseous N_2_ to the FR. Only fuel-N should be present in the FR. Otherwise, the chemistry and equilibrium in the NOx formation will be affected by the presence of N_2_.

The evolution of fuel-N has been evaluated in *i*G-CLC with Fe ore^47^ and Mn ores,^48^ as well as in CLOU with Cu34Mn66 ^47^ and Mn66FeTi7.^56^ Pine wood, olive stone, and almond shell were used as fuels. In general, fuel-N is evolved in the FR almost completely due to the high fuel conversion achieved in this reactor. Thus, no issues related to NOx emissions to the atmosphere would be expected. At the typical operating temperatures in CLC (>850 °C), fuel-N in the FR was mainly converted to N_2_, with the presence of some NOx but with N_2_O not being observed. Some differences have been found when comparing results under *i*G-CLC and CLOU modes; see Figure 20.^47^ In *i*G-CLC, a reducing atmosphere is found in the FR due to the presence of unburnt products, which favored the fuel-N conversion to N_2_, and the low presence of NOx. To achieve CLOU conditions with the Cu34Mn66 oxygen carrier, a temperature of 850 °C is desired in order to have oxidizing conditions in the FR. In this case, the conversion of fuel-N in NOx is similar to that in the *i*G-CLC mode. However, NOx may be reduced as temperature decreases due to the lower oxygen uncoupling relevance.

**Figure 20:**
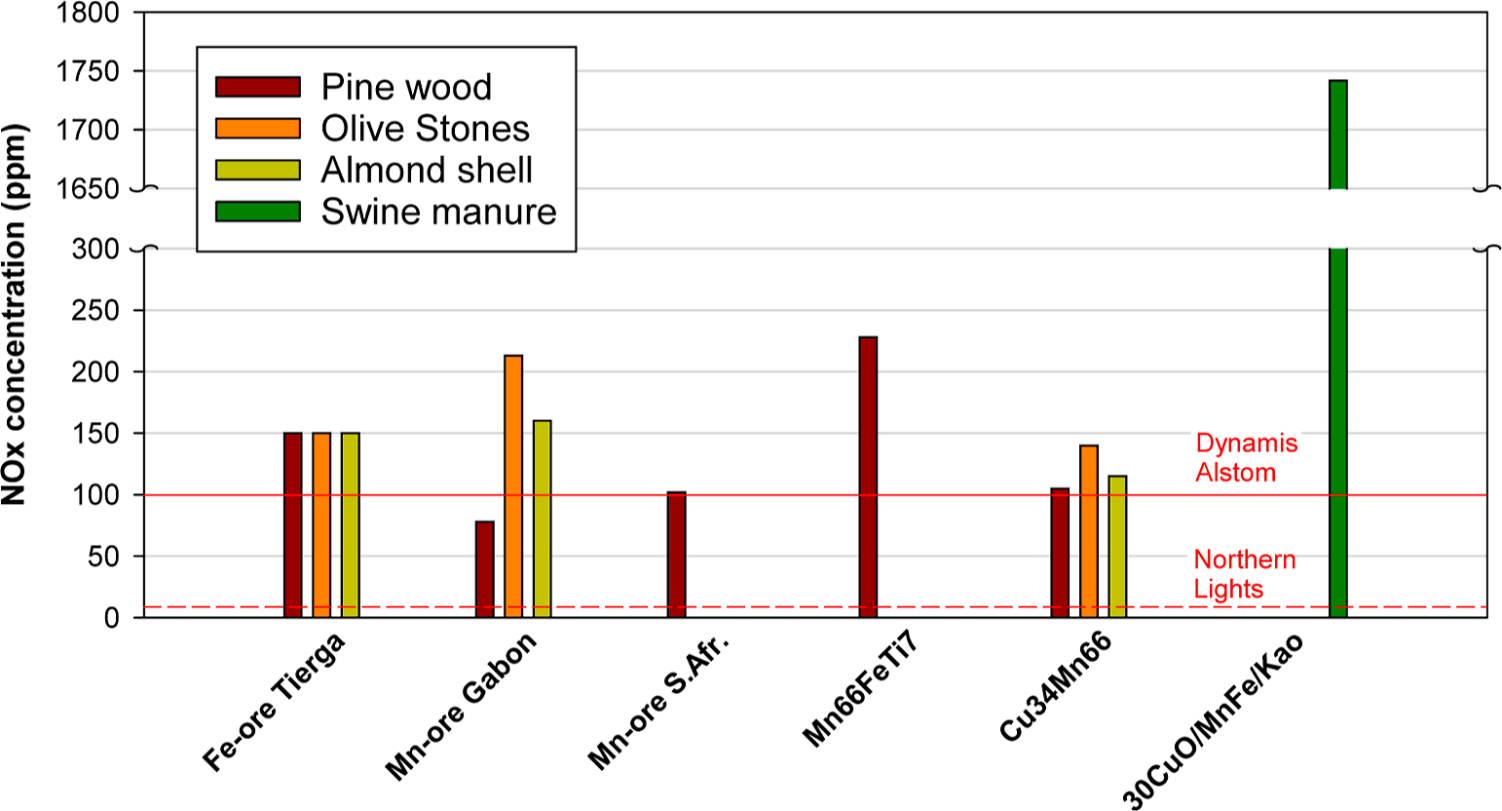
NOx concentration in the CO_2_ stream from the FR with the oxygen carriers and biomasses used in the 0.5 kW_th_ CLC unit.

The presence of NOx in the CO_2_ stream should be addressed considering its final use, which may be different depending on if it is used in the industry (e.g., food industry), used as feedstock to produce chemicals or fuels (e.g., methanation), used in greenhouses to enhance plant growth, or permanently stored in geological formations. In any case, the specifications for the CO_2_ quality must be guaranteed by taking the necessary measures. In the case that Bio-CLC is considered as a NET technology for CRD, transportation and storage of CO_2_ as a supercritical fluid has a limit of 100 ppm of NOx.^77^ The value achieved in the tests here presented often exceed this limit by 50–100%, although lower values were obtained in some cases.^47,48,56^ Recently, the Northern Lights Project reduced considerably this value to 10 ppm of NOx for CO_2_ transportation in single-phase liquid to avoid the presence of noncondensable gases.^78^

In the evaluation of fuel-N, relevant are the recent results presented for the combustion of swine manure by CLOU with the 30CuO/MnFe/Kao oxygen carrier.^54^ Swine manure has a high N content—see Table 3—which affects its conversion in the FR. This nitrogen is very volatile and was emitted exclusively in the FR regardless of the char conversion. In this case, the main compound in the combustion gases was also N_2_. However, the fraction of N converted into NOx varied from 2 to 8%, with the highest values as the temperature increased from 800 to 950 °C. Also, lower NOx amounts were found in H_2_O compared to tests fluidizing with CO_2_. In addition, a small fraction of unconverted NH_3_ (0.1–0.5%) was detected. The NOx concentration in the CO_2_ stream took values in the 1045–5030 ppm interval, well above the limit considered for the CO_2_ transport. Nevertheless, more than 95% of fuel-N in swine manure was inertized in the form of N_2_, which represents a benefit regarding the environmental problem that this waste presents.

### 4.5 Oxygen Carrier Issues with Biomass

In Bio-CLC, the biomass is fed to the FR, and therefore, it is mixed with the oxygen carrier particles. This fact causes some elements present in ash from the fuel to interact with compounds in the oxygen carriers. This topic has been the subject of study in multiple works dedicated to it. Accelerated ash–oxygen carrier interaction has been studied in batch reactors. For example, K and Na may either diffuse inside oxygen carrier particles or remain as an external layer covering the particles with Fe-based oxygen carriers^80^ or Mn ores^81^ which may cause bed agglomeration, although changes in the reactivity have not been observed.^82^ Some interaction has been also observed with alkaline-earth compounds, such as Mg and Ca.^83^ With CuO-based materials, special interaction was observed with Na, affecting the reactivity and/or regeneration capability,^84^ but no other issues may be expected with ash from biomass.^85^ Also, the presence of Cl may deactivate a fraction of the active phase, but it may be minimized in the presence of steam.^86^

However, results on the interaction in real operating conditions are scarcer, and they have the main drawback of the relatively short operation time compared to the expected lifetime of the oxygen carrier. Some conclusions may be extracted from tests burning biomass in a fluidized bed replacing the bed material by an oxygen carrier, namely, the oxygen carrier-aided combustion, at the 12 MW_th_ scale with ilmenite^87^ and Fe-based waste.^88^ Also, some information is available for a CaMn-based perovskite used in Bio-CLC at the 80 kW_th_ scale.^34^ In general, although some elements were accumulated on the oxygen carrier particles, no detrimental effects were observed regarding agglomeration or reactivity related to the ash–oxygen carrier interaction.

Anyway, some interesting results may be extracted from the operational experience in CLC units with biomass. In no case agglomeration of the oxygen carrier material was observed due to the presence of ashes in the bed. This would be expected with pine wood, which has a very low ash content; but avoidance of agglomeration issues has been also observed for the rest of the biomass including swine manure. In addition, there is no evidence that the ash–oxygen carrier interaction could negatively affect the oxygen carrier reactivity. In fact, ash elements have not been found in Fe-ore Tierga, 60CuO/MgAl_2_O_4_, and 30CuO/MnFe. In the case of 30CuO/MnFe/Kao, some alkali were found in particles associated with the presence of kaolin.

Some degradation of the mechanical properties has been observed with the use of the oxygen carrier. In general, the porosity of the particles increases during its use in the CLC units, which is accompanied by a decrease in the crushing strength and an increase in the attrition rate. This effect has not been directly related with the interaction with ash particles, but it has been related to the chemical stress due to phase changes in the solid during redox cycles.^60^ So, metal migration and/or cracks due to changes in the crystalline structure have often been observed. This issue is considered to be a minor problem for low-cost materials. However, the lifetime of synthetic particles for CLOU should be improved. Thus, the addition of kaolin to the 30CuO/MnFe material improved its mechanical performance.^52^ However, the long-term operation of the materials should be performed to gain confidence on the use of synthetic oxygen carriers during the scale-up of the CLOU process.

In addition, the ash should be drained from the bed to avoid its accumulation. On the one hand, a fraction may be recovered as fly ash, which may be mixed with fines coming from the abrasion/fragmentation of oxygen carrier particles. On the other hand, no-elutriated ash particles should be removed from the bed, which can be mixed with oxygen carrier particles. In any case, the oxygen carrier must not present toxicity or risk for the environment in order to avoid the contamination of ash by the presence or lixiviation of dangerous products. Considering the oxygen carriers used in the 0.5 kW_th_ CLC unit with biomass, a previous work showed that Fe-ore Tierga was classified as stable nonreactive hazardous waste, and the drained ash may be disposed in a landfill for nonhazardous residues.^89^ In addition, valuable materials in the oxygen carrier may be recovered from ashes in the case that a magnetic oxygen carrier is used,^51^ such as it is the case of Mn66FeTi7 and Cu-based materials supported on MnFe mixed oxides.

### 4.6 Applications of the Operational Experience to the Design of a Bio-CLC Unit

The operational experience here presented has shown the potential of the *i*G-CLC and CLOU processes to burn biomass with CO_2_ capture in two CLC units: (1) 0.5 kW_th_ unit with the FR being a bubbling fluidized bed and (2) 50 kW_th_ unit with the FR being a circulating fluidized bed operating in the turbulent regime. In addition to the results presented about the CO_2_ capture and combustion efficiency in both units with biomass, the following useful information may be extracted to be considered for a suitable design of the FR:

•Char conversion rate, (−*r*_C_): to properly calculate the char conversion rate, it is required to previously calculate the mean solid residence time of char particles in the FR, *τ*_char_, the char concentration in solids exiting the FR, and the mass of char in the FR.^33,45^ However, after some algebra, it was found that the char conversion rate may be easily calculated by knowing the char conversion and the mean residence time of solids in the FR:

11 $$\left(-r_{C}\right)=\frac{1-\frac{F_{char,AR}}{F_{char,f}}}{\tau_{char}}=\frac{X_{char}}{\tau_{char}}=\frac{X_{char}}{\tau_{OC}\left(1-X_{char}\right)}$$

Once the char conversion rate is known, it is possible to estimate the char conversion under a determined solids circulation rate (*ṁ*_OC_) and solids inventory in the FR (*m*_FR_) and then estimate the CO_2_ capture rate by eq 9. Also, the separation efficiency of a CS may be included in this calculation.^33,42^

•The rate of oxygen transference in the FR: it may show if the oxygen carrier has some limitation transferring oxygen in the FR.^61^ The rate of oxygen transference will affect the combustion efficiency in the FR, i.e., the presence of unconverted products from the FR. For example, in CLOU, it was estimated that for highly reactive fuels, the minimum solid inventory in the FR is determined by that required to achieve complete combustion, which in turn is determined by the rate of oxygen transference. On the contrary, the solids inventory with low reactive fuels will be determined by that required to achieve high CO_2_ capture.^60^
•The fraction of unconverted gases from the FR: unburnt products may come from volatile matter and char gasification. Some attempts have been made to estimate the oxidation degree of volatiles and gasification products, for example, based on the methane conversion^37^ or by fitting oxygen demand values for different char conversions.^20^ The relevance of unconverted compounds from volatiles or char gasification is a function of the FR fluid dynamics. While most of unburnt products in a bubbling FR come from volatile matter,^90^ unconverted products from char gasification may be relevant in a circulating fluidized bed.^20^

A tentative design of the FR may be proposed from results in the CLC units, and its performance may be estimated by evaluating the CO_2_ capture rate (η_CC_) and the oxygen demand (Ω_OD_). For example, Figure 21 shows the estimated CO_2_ capture as a function of *ṁ*_OC_ and *m*_FR_ with the calculated value of the char conversion rate for olive stones at 950 °C, being (−*r*_C_) = 0.032 s^–1^ for both the 0.5 and 50 kW_th_ units. Thus, the contour lines for different η_CC_ values are valid for both bubbling and circulating fluidized beds. The CO_2_ capture increases with the solids inventory and by decreasing the solids circulation rate, i.e., as τ_char_ increases. The minimum CO_2_ capture rate corresponds to the fraction of carbon evolved in volatile matter, while it increases as more char is gasified. Considering Fe-ore Tierga as the oxygen carrier, the minimum solids circulation rate to transport the stoichiometric oxygen for the biomass combustion, i.e., for ϕ = 1, is 3 kg/s per MW_th_. In this case, a value of η_CC_ = 97% would be expected with *m*_FR_ = 1000 kg/MW_th_. However, the minimum circulation rate is lower for oxygen carriers with a higher oxygen transport capacity, *R*_OC_, such as Mn ores—see Table 4—which would allow for higher η_CC_ values.

**Figure 21:**
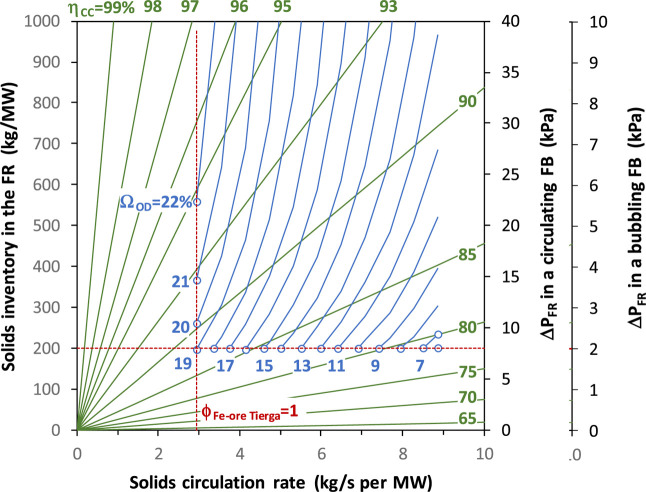
Estimated CO_2_ capture rate (η_CC_) and oxygen demand (Ω_OD_) for the combustion of olive stones using Fe-ore Tierga as the oxygen carrier as a function of the solids circulation rate and the solids inventory in the FR. The corresponding pressure drop for the FR being either a bubbling (*S*_FR_ = 1 m^2^/MW) or circulating fluidized bed (*S*_FR_ = 0.25 m^2^/MW) is also shown. The oxygen demand values are estimated from results in the 20 kW_th_ CLC unit, with the FR being a circulating fluidized bed.^57^

Figure 21 also shows the estimated oxygen demand values from results obtained at the 20 kW_th_ CLC unit.^57^ A minimum solids inventory of 200 kg/MW_th_ is assumed for a proper operation of the fluidized bed, and it was observed that the oxygen demand significantly increases below this value; see Figure 17. Thus, with this solids inventory, the oxygen demand decreases from 19% to 6% when the solids circulation rate increases from 3 to 9 kg/s per MW_th_. At the same time, the CO_2_ capture decreased from 88% to 78% due to the decrease in the solids residence time in the FR. In order to increase the CO_2_ capture, the amount of solids in the FR may be increased to allow a higher char conversion. The increase of gasification products in gases causes the oxygen demand to increase with the solids inventory in the FR, as was experimentally observed; see Figure 17. Therefore, there should be a trade-off between achieving high CO_2_ capture and low oxygen demand values. A CO_2_ capture rate of η_CC_ = 90% may be achieved with a solids inventory in the FR of *m*_FR_ = 500 kg/MW_th_ and 6 kg/s per MW_th_ (ϕ = 2), which may be considered suitable operating conditions for CLC with solid fuels.^91^ In this case, the estimated oxygen demand is Ω_OD_ = 15%.

Olive stone has low reactivity compared to other biomasses such as pine wood, almond shell, or wheat straw.^46^ Therefore, higher CO_2_ capture rates would be expected for other biomasses. In any case, the CO_2_ capture may be increased by using an effective carbon separation system in the solid stream from the FR to the AR. For example, a CS is able to elutriate light and small fuel particles—e.g., powdered coal—from denser oxygen carrier particles.^37^ However, the CS was not suitable for millimetric particles, such as those corresponding to biomass, and alternative concepts will be required.^38^ In addition, the CO_2_ capture may be improved by further increasing the solids inventory in the FR. However, this option affects the pressure drop in the FR, Δ*P*_FR_. Considering the gas velocity in the FR to operate it as a circulating fluidized bed, a suitable value for the FR cross section is *S*_FR_ = 0.25 m^2^/MW,^91^ which corresponds to Δ*P*_FR_ = 20 kPa for *m*_FR_ = 500 kg/MW. This pressure drop would be lower if the FR was operated in the bubbling regime, which allows a cross section of *S*_FR_ = 1 m^2^/MW,^64^ decreasing the corresponding pressure drop to Δ*P*_FR_ = 5 kPa. However, it would be expected that the oxygen demand would be increased by 25–30% compared to that in a circulating fluidized bed; see Figure 17.

## 5 Challenges and Future Trends

The information shown in this work, together with the operational experience achieved by other researchers in the field, may be useful for the future development of the Bio-CLC process. For example, additional and relevant information may be also acquired from operational experience of a 200 kW_th_ at the University of Utah.^92^ They found that the addition of a Cu-based material with oxygen uncoupling capability to ilmenite improved the combustion performance, but also, it can make plant operation easier under autothermal conditions. Thus, the FR with ilmenite is endothermic, but it may be exothermic in CLOU which simplifies energy integration between the FR and AR.

In general, operating or design conditions have been identified to maximize either CO_2_ capture or combustion efficiency. However, it has been difficult to maximize both parameters at once. Thus, several challenges may be identified, which mark the path to follow in future development of the Bio-CLC technology.

Some relevant challenges to be addressed are as follows:

•Being able to achieve high CO_2_ capture rates regardless of the kind of biomass: flexibility in biomass used as fuel can reinforce the use of fluidized beds in Bio-CLC. In most of the cases, using a CS is not suitable for this challenge with biomass, although it was promising for powdered coal. Therefore, a proper design of the FR would be required to allow high-enough values of the mean residence time of char particles in the FR to achieve high char conversion values and CO_2_ capture rates.
•Improving the combustion efficiency at high CO_2_ capture rates: the FR design should consider the conversion of both volatile matter and gasification products from biomass. Volatiles are more poorly converted in a bubbling fluidized bed, while gasification products become relevant in a circulating fluidized bed.
•Finding suitable and reliable oxygen carrier materials for the scale-up of the Bio-CLC process: the state of the art shows that ilmenite may be selected as a reference material for scaling up and long-time operation in demonstration plants. However, a challenge is to increase the portfolio of suitable materials for this technology.

The most direct way to increase the CO_2_ capture rate is to increase the residence time of char particles in the FR. If the implementation of a CS is considered for that, its concept and design must be rethought for the relatively big size of biomass particles. Thus, its design should be different from that usually adopted to separate small char particles coming from powdered coal from dense oxygen carrier particles. Alternatively, the residence time of char particles may be done in the bubbling regime by increasing the solid inventory in the FR without an excessive increase of its pressure drop. In this case, a design of the FR in the bubbling regime would be advised even at the cost of having worse combustion. In a circulating fluidized bed, the char conversion was evaluated considering the mean residence time of the oxygen carrier particles, which is known; e.g., see Figure 12. But the solid residence time of char in the FR may be different to that of the oxygen carrier due to different fluid dynamic properties. Thus, a dedicated study would be required to evaluate the char conversion in the FR. Designs trying to make it difficult for char particles to exit the cyclone would be useful to increase their conversion and the CO_2_ capture. Also, high FR temperatures or using materials with oxygen uncoupling capabilities will help achieving such purposes.

The achievement of a CO_2_-rich stream that is ready for compression and transport should be accomplished. The CLOU process with a bubbling FR allowed achieving complete combustion with high-enough solid inventory, and additional measures would be not advised. But in general, a CLC unit should be coupled to a gas processing system, including an oxygen polishing step downstream the FR which should be designed and evaluated in order to oxidize unconverted gases. But also, the performance of converting tar compounds and solid carbon in gases from the FR, its effect on pollutants (such as NOx or SOx), and the presence of oxygen in excess remaining as O_2_ in the CO_2_ stream, as well as fines (both from ash and oxygen carrier), should be considered.

The knowledge of the origin of main unconverted products from the FR will allow us to apply determined measures to minimize the oxygen demand of the Bio-CLC process. The quick evolution of volatile matter in the FR with biomass causes volatiles to evolve in plumes with low efficiency of contact with the oxygen carrier particles, which are mostly in the emulsion phase. To improve the gas–solid contact of volatile matter in the dense phase of a fluidized bed, the use of a volatile distributor^93^ or the introduction of foreign, big, and nonfluidizable particles in the bed^94^—the so-called confined fluidization—has been proposed.

Char gasification in the riser would hinder complete oxidation in the lean phase, even in CLOU mode. In order to improve the combustion degree in the riser, the implementation of some constrictions in the riser wall has been proposed,^40^ as well as promoting a counterflow of solids in the FR riser by returning the solids at an intermediate height.^95^ In this way, the circulation of descending solids through the riser is modified, and solids are accumulated on each constriction, modifying the solid distribution in the circulating fluidized bed. In fact, the use of ring-type internals improved the combustion efficiency of coal in the 50 kW_th_ CLC unit by 20%.^39^ Then, this kind of FR design was evaluated with biomass at the 80 kW_th_ scale at the Vienna University of Technology with ilmenite^96^ and a synthetic CaMn-based perovskite.^34^ With ilmenite, the combustion of bark pellets gave an oxygen demand of Ω_OD_ = 18%, which could be decreased to Ω_OD_ = 8% by adding limestone particles to the ilmenite. The improvement was due to an increase of the solid circulation rate and temperature due to lower density of limestone particles, as well as the catalytic effect of CaO as it was shown previously with coal.^97^ The CaMn-based perovskite showed oxygen uncoupling capability, which reduced the oxygen demand to Ω_OD_ = 0.4%. This fact suggests that the use of ring-type internals may be of great interest to reduce the oxygen demand in the CLOU process when the FR is designed as a circulating fluidized bed reactor. However, this solution needs to be rethought or adapted in the case of applying it to large installations with a large cross-sectional area.

In addition, some solutions may be applied regardless of the FR design to improve the combustion efficiency, thus avoiding or minimizing the oxygen polishing requirements. The use of a secondary FR is an option that may allow a high reduction of the oxygen demand, as it has been theoretically evaluated for the combustion of pine wood with Fe-ore Tierga.^45^ The advantage of this option is that the behavior of the secondary FR is that of a CLC unit with gaseous fuels which is easier to operate and design.^64^ However, a proper integration in the CLC unit should be done, allowing the solid circulation between all of the reactors. One option to facilitate this integration is the splitting of the FR in two zones separated by perforated plates or bubble caps: in the bottom, the biomass is fed and partially converted by the oxygen carrier, while in the upper part, a fluidized bed ends the combustion of gases coming from the bottom zone.^98^ Using this FR concept, the oxygen demand could be decreased by 35% burning sewage sludge^99^ or by 66% burning wood^100^ at the 1 and 25 kW_th_, respectively. Improved results are expected by stacking more stages in the so-called tower fluidized bed.^101^ Alternatively, the CS of a 50 kW_th_ CLC unit has been used as a primary FR, being the former FR in Figure 5 now used as a secondary FR.^102^ In this case, the oxygen demand decreased by 24% burning coal.

The recirculation of flue gases from the FR is another alternative to reduce the oxygen demand of the Bio-CLC process.^103^ The gas recirculation was simulated in the 0.5 kW_th_ CLC unit burning pine wood with the manganese ores described in Table 1, showing a decrease of the oxygen demand up to 80%.^48^ In addition, it has also been proposed to recirculate only unburned products (e.g., H_2_, CO, or CH_4_) by separating them from CO_2_ and H_2_O.^28^ This option virtually eliminates the oxygen demand requirements because CO_2_ and H_2_O are the only eventual combustion products. However, N_2_ from the fuel remains mixed with unconverted products, and it should be purged to prevent its accumulation in the system. In the purge stream, some H_2_, CO, and CH_4_ are present. To avoid losses in the combustion efficiency, these products are proposed to be burnt in the AR. Then, CO_2_ is emitted from this reactor, decreasing the CO_2_ capture rate of the CLC process.

In addition, the long-term operation of Bio-CLC under relevant industrial conditions should be performed to evaluate the durability of the oxygen carrier particles. Promising behavior is shown for some low-cost materials, such as ilmenite or Fe ore. However, research is still required to produce synthetic materials with a high-enough lifetime for the scale-up of the CLOU process, mainly focused on highly reactive Cu-based materials. Thus, there is an important field of study and range of improvement in the development of oxygen carriers to be used in Bio-CLC, especially those synthetic carriers with oxygen uncoupling capability.

To a certain extent, a relatively low lifetime due to a loss of properties is acceptable for low-cost materials from natural ores or industrial wastes. However, the lifetime of synthetic particles must be improved, as they end up degrading after several hours of operation in aspects such as the mechanical strength, increased porosity, phase change, or loss of regenerability. Based on thermodynamic and kinetic considerations for the oxygen uncoupling reaction, materials based on CuO and Mn mixed oxides are the most promising for use with biomass. Degradation has been observed for particles prepared by granulation, e.g., CuO-based,^51,53^ CuMn mixed oxides,^55^ MnFe mixed oxides,^56^ and CaMn-based perovskites.^34^ However, some materials based on Ni, Cu, and Fe prepared by impregnation have shown high durability with both gaseous and solid fuels.^104^ Among them, although without oxygen uncoupling capability, a Cu-based material impregnated on Al_2_O_3_ has shown interest for its use with solid fuels with an estimated lifetime of 8000 h.^105^ In addition, the high reactivity of this oxygen carrier has made it possible to significantly reduce the oxygen demand up to 4.4% in a bubbling FB.^100^ Furthermore, it was reduced to Ω_OD_ = 1.5% by using a secondary FR. In addition, recent results show that its use with swine manure also reduced the oxygen demand to 2% in the 0.5 kW_th_ unit.^106^

Note that the observed degradation of the oxygen carrier particles is related to the chemical and thermal stress that the particles experience during their use in a CLC unit. However, longer operation is required to evaluate the aging effect of the ash interaction with the metal oxides in the oxygen carrier particles. To date, operation for 100 h or less has shown some accumulation of elements from the ash in the oxygen carrier particles but without showing relevant negative effects, such as deactivation or agglomeration.

Anyway, the issue of ash accumulation in the bed material should be also assessed.^89^ Thus, a fraction of the oxygen carrier may be found mixed with the ashes extracted from the CLC unit. Their recovery is desirable if they are expected to have value, both economically and for reuse. In this sense, the development of particles with magnetic properties facilitates oxygen carrier recovery from the ash particles. In the lab, tests to evaluate the capability to recover oxygen carrier particles from ash are performed at room temperature. In the industrial process, the optimum temperature to perform this separation may be higher. This temperature should be evaluated considering the Curie temperature at which magnetic properties are lost, which is in the 300–450 °C interval for ferrites including manganese and/or copper.^107^ Because the solid stream should be cooled before the magnetic separation, the thermal integration of the ash purge stream in the CLC unit should be evaluated.

New materials may be developed considering the huge work done exploring multiple mixed oxide compounds,^108^ including materials with oxygen uncoupling capability. Specifically, these materials must show resistance to the interaction with ash elements, in addition to suitable thermochemical properties to be used in Bio-CLC and mechanical integrity during thousands of redox cycles in a CLC unit. Preservation of the mechanical integrity highlights the development of new oxygen carriers based on low-entropy materials,^26^ which can be of great interest for the development of the CLOU process in the future.

## 6 Conclusions

The outstanding properties of unmixed combustion of biomass by chemical looping (Bio-CLC) make this process an efficient way to produce energy by thermochemical conversion, preventing the CO_2_ and NOx emissions to the atmosphere. Results of burning different biomasses with several oxygen carriers under *i*G-CLC and CLOU and in two units (bubbling the FB in the 0.5 kW_th_ unit and circulating the FB in the 20–50 kW_th_) are presented. Then, a comprehensive evaluation and comparison of the CLC performance is done regarding the process (*i*G-CLC vs CLOU), the fluidization regime (bubbling vs circulating), the oxygen carrier, or the fuel used. The most relevant conclusions are

- Biomass combustion is not complete in *i*G-CLC operation, but the oxygen demand decreases using more reactive oxygen carrier materials, this factor being relevant in the selection of a suitable oxygen carrier with biomass. In CLOU, complete combustion in the FR was generally achieved for different biomasses in the 0.5 kW_th_ unit, i.e., the FR being a bubbling FB. A less effect of the solids inventory on the combustion efficiency was observed, which was explained on the basis of the less efficient accumulation of solids in the dense bed. However, increasing the solids inventory is useful to achieve higher CO_2_ capture rates.
- Differences between the fluidization regime of the FR on the CLC performance are highlighted; see the discussion on Figures 12b and 17b. The most relevant difference was an improvement of the combustion efficiency by using a circulating FB in *i*G-CLC mode, where the conversion of volatile matter was improved. But unburnt products appeared in the CO_2_ stream from the 50 kW_th_ unit (operated as a circulating FB) in CLOU mode, which were nonexistent in the smaller unit in the bubbling regime. In this case, it is believed that inefficient conversion of volatile matter both in the dense and in the lean phase in the riser is a primary source of unburnt products due to a low temperature in the FR.
- CO_2_ capture higher than 95% may be achieved with wood due to the fast gasification both in H_2_O and CO_2_. Therefore, the FR may be fluidized by recycled CO_2_, reducing in this way the energy duty of the CLC plant compared to the use of steam. The fluid dynamics of the FR did not affect the char gasification rate and the CO_2_ capture. The effect of the fixed carbon content and fuel reactivity on the CO_2_ capture was described in detail; see Section 4.1, and specifically, Figure 15. The use of biomasses with lower reactivity, such as olive stones, limits the attainable CO_2_ capture to ∼90% in a circulating fluidized bed. Higher CO_2_ capture values would require higher amounts of solids in the FR with the corresponding increase in the pressure drop in the reactor; at least the FR was conceived as a bubbling fluidized bed. Higher CO_2_ capture rates may be achieved in CLOU by having two ways to convert solid carbon in char in parallel, namely, gasification by the fluidizing gas (preferably recirculated CO_2_) and combustion by released O_2_ via oxygen uncoupling.
- Suitable conditions to perform a reliable comparison are determined; e.g., see Figure 16. Thus, oxygen demand values in the 10–15% interval were obtained under optimum operating conditions, considering also the suitable conditions to maximize the CO_2_ capture. A clear and concise explanation of the effect of char conversion on combustion efficiency was presented, see Section 4.2 and specifically, Figure 17. Thus, the achieved combustion efficiency values should be discussed joined to the current char conversion. Namely, sometimes high combustion efficiency values may be achieved but at conditions having low char conversion values. The combustion efficiency could severely decrease at relevant CLC conditions where high char conversion values are obtained to achieve high CO_2_ capture rates.
- Although low NOx and tar content is observed in the CO_2_ stream, some measures would be required to minimize the concentration of these compounds for CO_2_ use, transport, and storage. Anyway, additional measures to avoid polluting emissions into the atmosphere are not required, as low NOx in the air exhaust stream is anticipated.
- A wide field of research has been identified for the future development of new oxygen carriers for Bio-CLC, as well as finding optimum operating and design conditions to maximize both combustion efficiency and CO_2_ capture rate.

## Abbreviations

AR: air reactor
ARX: X^th^ Assessment Report
BECCS: bioenergy with carbon capture and storage
Bio-CLC: biomass-based chemical looping combustion
CCS: carbon dioxide carbon capture and storage
CDR: carbon dioxide removal
CLaOU: chemical looping assisted by oxygen uncoupling
CLC: chemical looping combustion
CLG: chemical looping gasification
CLOU: chemical looping with oxygen uncoupling
COP: climate change conference
CS: carbon stripper
EU: European Union
FB: fluidized bed
FR: fuel reactor
GHG: greenhouse gas
ICB-CSIC: Instituto de Carboquímica-Consejo Superior de Investigaciones Científicas (Spanish National Research Council)
*i*G-CLC: in situ gasification chemical looping combustion
IPCC: Intergovernmental Panel on Climate Change
KPIs: key performance indicators
LHV: lower heating value
NETs: Negative Emissions Technologies
OC: oxygen carrier
OCAC: oxygen carrier-aided combustion
PFR: pine forest residue
R&D: Research and Development
STP: standard temperature and pressure
UN: United Nations
*f*_C_: fraction of carbon in fuel (−)
*f*_C,fix_: fraction of carbon in fuel as fixed carbon (−)
*f*_C,vol_: fraction of carbon in fuel in volatile matter (−)
*F*_char,AR_: flow of carbon in char to the air reactor (kg/s)
*F*_char,f_: flow of carbon in char fed to the fuel reactor (kg/s)
*ṁ*_f_: mass flow of the solid fuel (kg/s)
*m*_FR_: specific inventory of solids in the fuel reactor (kg/MW_th_)
*ṁ*_OC_: solid circulation rate (kg/s)
*ṁ*_OCst_: specific stoichiometric flow of the oxygen carrier to supply the oxygen required to oxidize the fuel to CO_2_ and H_2_O, considering the oxygen carrier is fully oxidized (kg/s per MW_th_)
*R*_OC_: oxygen transport capacity of the oxygen carrier (−)
(−*r*_C_): char conversion rate (s^–1^)
*S*_FR_: cross section of the fuel reactor (m^2^/MW_th_)
*T*_FR_: temperature in the fuel reactor
*X*_char_: char conversion in the fuel reactor (−)
*X*_char,eff_: effective char conversion in the fuel reactor (−)
*X*_f_: fuel conversion (−)
*χ*_oo_: oxide oxygen fraction (−)
Δ*P*_FR_: pressure drop in the fuel reactor (Pa)
ϕ: oxygen carrier to fuel ratio (−)
*η*_CC_: CO_2_ capture rate (−)
*τ*_char_: mean residence time of char in the fuel reactor (s)
*τ*_FR_: mean residence time of the oxygen carrier in the fuel reactor (s)
Ω_f_: oxygen demand of the solid fuel (kg O_2_/kg fuel)
Ω_OD_: total oxygen demand of the CLC process (−)
